# On photokinetics under polychromatic light

**DOI:** 10.3389/fchem.2024.1367276

**Published:** 2024-04-22

**Authors:** Mounir Maafi

**Affiliations:** Leicester School of Pharmacy, De Montfort University, Leicester, United Kingdom

**Keywords:** polychromatic light, photokinetics, Φ-order kinetics, actinometry, reactivity–wavelength range, Runge–Kutta

## Abstract

Since the dawn of photochemistry 150 years ago, photoreactions have been conducted under polychromatic light. However, despite the pivotal role that photokinetics should naturally play for such reactive photosystems, the literature lacks a comprehensive description of that area. Indeed, one fails to identify explicit model integrated rate laws for these reactions, a characteristic type for their kinetic behavior, or their kinetic order. In addition, there is no consensus in the community on standardized investigative tools to evaluate the reactivity of these photosystems, nor are there venues for the discussion of such photokinetic issues. The present work is a contribution addressing some of these knowledge gaps. It proposes an unprecedented general formula capable of mapping out the kinetic traces of photoreactions under polychromatic light irradiation. This article quantitatively discusses several reaction situations, including the effects of initial reactant concentration and the presence of spectator molecules. It also develops a methodology for standardizing actinometers and defines and describes both the spectral range of highest reactivity and the photonic yield. The validity of the model equation has been proven by comparing its results to both theoretical counterparts and those generated by fourth-order Runge–Kutta numerical calculations. For the first time, a confirmation of the Φ -order character of the kinetics under polychromatic light was established.

## 1 Introduction

Green chemistry, sustainability, atom-, and circular economies are among the main scientific challenges of the twenty-first century for which photochemistry is expected to play an essential role. Indeed, the availability and ubiquity of cheap sunlight, as well as the potential for harvesting it in many innovative ways and the accessibility to a variety of cheap LED lights, have promoted photochemistry to be viewed as an important gateway toward bridging the gaps between the current situation and future aspirations. A large variety of photoprocesses and photosystems have already been studied for that purpose, including solar thermal energy storage (Gimenez-Gomez et al., 2022), artificial molecular machines (Andreoni et al., 2021), photomicroreactors powered by direct sunlight (Cambié et al., 2019), photoresponsive materials (Li et al., 2019), water-splitting (Han et al., 2014), pharmaceuticals (Holland et al., 2020; Le Basle et al., 2020), manufacturing chemicals with light (Poliakoff and George, 2020), flow photochemistry (Williams and Kappe, 2020; Buglioni et al., 2022), micro and mesostructured photoreactors (Kayahan et al., 2020), environmental impact (Vione and Scozzaro, 2019), polymer mechanochemistry (McFadden et al., 2023), a variety of potential industrial applications (Griesbeck et al., 2012), and a range of flexible materials (Yamada and Yagai, 2019).

The investigations directed toward innovative processes have seldom used monochromatic light but are typically driven by natural or artificial polychromatic light. This type of light variably affects phototransformations due to the many parameters that impact photoreactivity. Better control, quantification, and reproducibility of such processes are, therefore, only possible with an adequate description of their photokinetics. Unfortunately, a standard and comprehensive photokinetic approach for reactions performed under polychromatic light has been lacking in the field (Mauser et al., 1998; Tonnesen, 2004; Griesbeck et al., 2012; Yamada and Yagai, 2019; Montalti et al., 2020). The kinetics of these photoreactions has no equivalent counterparts for the basic concepts of thermal chemical kinetics, such as established reaction kinetic orders and/or identified integrated rate laws. This situation might not seem awkward if we consider the complexity of the mathematical framework that describes the photokinetics of these reactions compared to that encountered in thermal chemical kinetics. Simply put, the rate laws of reactions under polychromatic light cannot be solved analytically. Therefore, neither the integrated rate laws nor the kinetic orders of these photoreactions are accessible. However, one must keep in mind that even if analytical solutions are not reachable for the rate laws of reactions driven by polychromatic light, it is not excluded that these reactions possess a specific kinetic behavior, unlike those observed for thermal reactions.

Despite these hurdles, several investigations have used different approaches to deal with the photokinetics of reactions exposed to polychromatic light. Thermal kinetic equations (characterizing zeroth,- first-, and second-order kinetics) have been applied to photoreaction data (Yassine et al., 2018; Trawinski and Skibinski, 2019; Dolinina et al., 2020; Grande et al., 2020). However, there is no simple interpretation of the obtained parameters (e.g., the reaction rate constant) given the complexity of the integro-differential rate law describing the real physical system (*vide infra* Eq. (1)). Another strategy used a power series expansion of the rate law to simplify the latter to an integrable form. This process is too approximative to deliver accurate results (usually using only the first-order expansion of the power series) (Tonnesen, 2004). One approach introduces a simplifying hypothesis to derive an explicit formula to fit the experimental data. The applicability of such an equation is, however, limited to the specific reactive system and experimental conditions it was developed for (Lehóczki et al., 2013; Józsa et al., 2018; Frigoli et al., 2020; Maafi and Al Qarni, 2022a; Maafi and Al Qarni, 2022b). A relatively popular method promotes the use of a high enough initial reactant concentration to ensure that the impinging polychromatic light is fully absorbed by the reactive medium (where the absorbance value of the reactive medium ought to be no less than 2) (Amouroux et al., 2019). In these conditions, the rate of the photoreaction is assumed to reach a limit (become constant), and its mathematical formulation resembles that of the zeroth-order thermal kinetics (Zepp, 1978; Stranius and Borjesson, 2017; Stadler et al., 2018). In another perspective, quantification of reactivity by initial reaction-velocity has been performed by using a polynomial probe function to fit the experimental kinetic traces (Pino-Chamorro et al., 2016).The fitting of photoreaction data has also been performed by applying numerical integration methods to the rate law, including the Runge–Kutta (Chernyshev et al., 2018) and Euler methods (Lente and Espenson, 2004; Michnyóczki et al., 2021). A discussion of the efficiency of numerical methods specifically for the elucidation of photokinetics showed the limitation of such methods owing to the occurrence of identifiability and/or distinguishability issues (Maafi, 2023). Overall, experimental data were well-fitted by the above individual techniques. One can also hint at some variability in the mathematical formulation of the rate law describing a photosystem under polychromatic light (Lente and Espenson, 2004; Aillet et al., 2014; Rochatte et al., 2017; Józsa et al., 2018), including the radiative transfer equation (Zalazar et al., 2005; Braslavsky et al., 2011).

The above information, succinctly reviewing the most commonly used photokinetic options for the treatment of reaction data obtained under polychromatic light-irradiation, strongly suggests the lack of a consensus over a standardized photokinetic approach and the absence of an explicit formula to map out the kinetic traces of such photoreactions. In addition to these knowledge gaps, there are also no clear quantitative descriptions for the effects of the initial concentration, spectator molecules, or incident radiation intensity on the reactivity of such photoreactions. These aspects are worth addressing in order to standardize photokinetic investigation. The present work contributes to that effort.

## 2 Experimentals

The $n_{sp}$ species of a given photoreaction mechanism are labeled $Y_{j}$ with the reactant being $X=Y_{0}$ , and the photoproducts taking $n_{sp}\geq j>0$ . The photoreaction mechanism involves $n_{\Phi }$ photochemical reaction steps where each particular species $Y_{j}$ is the start and/or end of $n_{\Phi_{j}}$ reaction steps. Each reaction step occurring between species $Y_{j}$ and $Y_{j^{\prime }}$ ( $Y_{j}\to Y_{j^{\prime }}$ or $Y_{j^{\prime }}\to Y_{j}$ with $j\neq j^{\prime }$ ) is characterized by a specific quantum yield, $\Phi_{Y_{j}\to Y_{j^{\prime }}}$ (or $\Phi_{{Y_{j}}^{\prime }\to Y_{j}}$ ). These quantum yields may or may not be wavelength-dependent. In addition, strictly speaking, $n_{\Phi_{j}}$ is a set of the $j^{\prime }$ indices of the species $Y_{j^{\prime }}$ linked to $Y_{j}$ (for instance, for the reaction $X⇋Y_{1}\to Y_{2}⇋Y_{3}$ , the sets for the species are $n_{\Phi_{0}}=1$ , $n_{\Phi_{1}}=0,2$ , $n_{\Phi_{2}}=1,3$ *,* $n_{\Phi_{3}}=2$ ).

**SCHEME 1:**
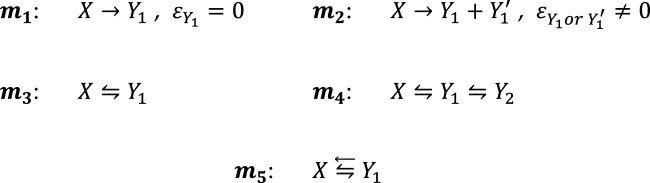
Most prominent examples of photoreaction mechanisms governing organic actinometers.

The phototransformation mechanisms studied in the present contribution are worked out from the Φ -shaped reaction mechanism described in a previous study (Maafi, 2023). Special attention is dedicated to the photomechanisms that operate most of the known organic actinometers (Kuhn et al., 2004; Braslavsky, 2007), as shown in Scheme 1.

In Scheme 1, the mechanisms correspond to the primary photoprocess with either a transparent photoproduct (***m***_***1***_, e.g., the diarylethene colored photoproduct only (Yamada and Yagai, 2019)) or an absorbing photoproduct, either or both $Y_{1}$ and $Y_{1}^{\prime }$ , (***m***_***2***_, e.g., uridine (Zhang et al., 1997)), the photoreversible reaction (***m***_***3***_, e.g., stilbenoids (Saltiel et al., 2022)), the doubly photoreversible reaction (***m***_***4***_, e.g., fulgides (Reinfelds et al., 2019)), and finally, reaction ***m***_***5***_, where the photoreversible reaction ( ⇋ ) induced by $∆\lambda_{1}$ -irradiation (e.g., UV) is overlayed by a back photoreaction (←) of the product only when concomitantly exposed to a radiation of a different wavelength range, $∆\lambda_{2}$ (e.g., visible). This reaction is typical of photochromic diarylethenes. Thus far, there is no published work on using the ***m***_***5***_-type reactions for actinometry, but the category is included here to extend the set of commercially available and potentially efficient actinometers to a group of photochromes offering a large dynamic range.

The kinetic traces corresponding to the temporal variation of the species concentration induced by a polychromatic light, $C_{Y_{j}}^{Lp,∆\lambda }\left(t\right)$ , have been calculated for each reaction by numerical integration. The fourth-order Runge–Kutta (RK) method used for that purpose is run by a home-made program on the Microsoft Excel VBA platform.

**FIGURE 1:**
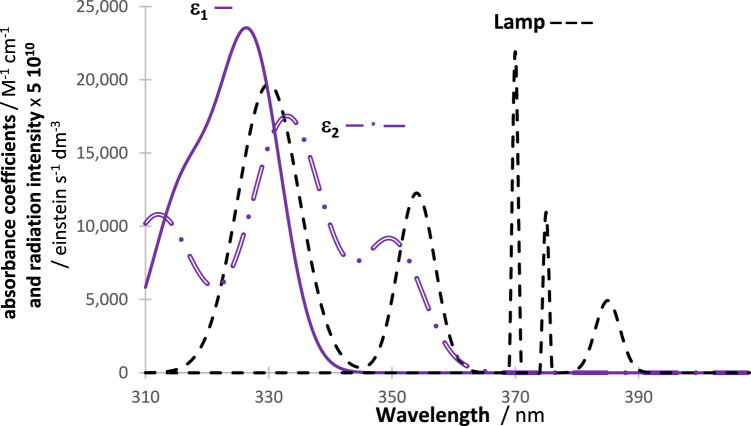
A general example of a disposition of an actinometer and investigated species absorption spectra (either $\varepsilon_{1}$ or $\varepsilon_{2}$ ) and a lamp profile.

The number of photons entering the reactor ( $P_{0}^{Lp,∆\lambda }=\sum_{\lambda_{a}}^{\lambda_{b}}P_{0}^{\lambda_{irr}}$ ) is measured as a sum of the number of photons delivered at each wavelength ( $\lambda_{irr}$ , in nm ). The detailed derivation of $P_{0}^{\lambda_{irr}}$ (expressed in $einstein\;s^{-1}\;dm^{-3}$ ) was previously provided (Maafi, 2023). This number of photons will naturally depend on both the span of wavelength ( $∆\lambda =\lambda_{b}-\lambda_{a}$ ) considered for the measurement and the profile of the lamp ( Lp ) used for irradiation. When it is determined by a physical actinometer, it may encompass the full wavelength span of the lamp emission, $∆\lambda_{Lp}$ . In contrast, a chemical actinometer will count only the incident photons (from the lamp) whose wavelengths belong to its absorption domain, $∆\lambda_{Act}$ . Similarly, the investigated species will respond to the light corresponding to its absorption spectrum, $∆\lambda_{sp}$ . The three wavelength spans can be smaller than, equal to, or larger than each other. In principle, the incident light is absorbed specifically in the overlap section of irradiation (lamp) and absorption (species) wavelengths, labelled as OSIA. Figure 1 shows an example of the layout of two absorption spectra and a lamp profile. These absorption spectra can each represent either an actinometer or an investigated species. The importance of the *OSIA* in actinometry will be reviewed in Section 3.9. Incidentally, the *OSIA* has sometimes been called the overlap integral (Tonnesen, 2004), a terminology that might lead to confusion because the rate is, itself, expressed by an integral (Eq. (1)), and hence, such a lack of specificity discourages its use.

The traces obtained by RK calculations (for reactant X and photoproducts $Y_{j}$ ) were fitted by the proposed general equation (*vide infra*, Eq. (6)). The fittings were performed by a Levenberg–Marquardt algorithm (LMA) provided by the curve fitting tool of R2020b MATLAB software. The assessment of the quality of the fitting was based on the squared correlation coefficients ( $r^{2}$ ) worked out from the linear plot of the RK-simulated and LMA-calculated trace of each species, the sum of squares error (SSE), and the root-mean-square deviation (RMSD) of the two data sets.

The number ( $i_{j}$ ) of mono-Φ-order terms $\left(\omega_{ij}^{∆\lambda }\;\mathrm{Log}\left(1+{cc}_{j}^{∆\lambda }e^{-\;k_{ij}^{∆\lambda }\;t}\right)\right)$ in Eq. (6) for a given species varies ( $n_{\Phi_{j}}\geq i_{j}\geq 1$ ) according to the values of intrinsic and extrinsic parameters or the type of the reaction (for instance, the trace of a photoreversible reaction whose total number of reaction steps is $n_{\Phi }=2$ is fully described by an equation holding a single mono-Φ-order term, that is, $i_{j}=1$ ).

In the present work, the rate laws correspond to a slab-shaped, vigorously, and continuously stirred reactive medium exposed to a collimated polychromatic light beam. These equations also consider that the concentrations of all species, at all times, fall within the linearity ranges of the respective calibration graphs of the species (e.g., the absorbance of the medium is less than a value of 0.5 at any reaction time and within the *OSIA*).

## 3 Results and discussion

### 3.1 The integro-differential equation of the rate law

The differential equation that describes the variation of the concentration, $C_{Y_{j}}^{Lp,∆\lambda }\left(t\right)$ (expressed in M or ${mol\;dm}^{-3}$ ), of a species with time ( t in s ), $r_{Y_{j}}^{Lp,∆\lambda }\left(t\right)$ (in ${{mol\;dm}^{-3}\;s}^{-1}$ ), must be expressed for each wavelength on the full polychromatic irradiation range, ∆λ (the actual *OSIA*). This means that an extra integral (and an extra integration variable dλ ) appears in the rate equation of a monochromatic light to relay the wavelength variation. Thereby, the left-hand side of the rate law is the classical differential $dC_{Y_{j}}^{Lp,∆\lambda }\left(t\right)/dt$ , and the right-hand side is an integral over λ (Eq. (1)). This equation can be amended by terms corresponding to reflection, scattering, and/or emission of light (Zalazar et al., 2005), but we consider here the simplest case where such contributions are negligible. So, for a given species $Y_{j}$ , the rate law is

$$r_{Y_{j}}^{Lp,∆\lambda }\left(t\right)=\frac{dC_{Y_{j}}^{Lp,∆\lambda }\left(t\right)}{dt}=\int_{\lambda_{a}}^{\lambda_{b}}\left(\sum_{j^{\prime }\;;\;j^{\prime }\neq j}^{n_{\Phi j}}\left(-\;\Phi_{Y_{j}\;\to \;Y_{j^{\prime }}}^{\lambda_{irr}}\;P_{a_{Y_{j}}}^{\lambda_{irr}}\left(t\right)+\Phi_{Y_{j^{\prime }}\;\to Y_{j}}^{\lambda_{irr}}\;P_{a_{Y_{j\prime }}}^{\lambda_{irr}}\left(t\right)\right)\right)\;d\lambda .$$ (1)

The sum under the integral accounts for the individual $n_{\Phi j}$ rate laws at each reaction step and at each individual irradiation wavelength ( $\lambda_{irr}$ ), expressed here as the product of the quantum yield ( Φ ) and the light absorbed ( $P_{a_{Y_{j\;or\;j^{\prime }}}}^{\lambda_{irr}}$ ) by the species at the start of that reaction step (the minus and plus signs indicate, respectively, depletion and formation of species $Y_{j}$ ). $P_{a_{Y_{j\;or\;j^{\prime }}}}^{\lambda_{irr}}$ is the time-dependent fraction of light absorbed specifically by $Y_{j\;or\;j^{\prime }}$ at time t and at the given $\lambda_{irr}$ ( $\lambda_{irr}=\lambda_{a},\ldots .,\lambda_{b}$ ), among all absorbing species present in the medium at time t and at that wavelength. It is expressed by (Maafi, 2023)

$$P_{a_{Y_{j\;or\;j^{\prime }}}}^{\lambda_{irr}}\left(t\right)=\frac{A_{Y_{j\;or\;j^{\prime }}}^{\lambda_{irr}}\left(t\right)}{A_{tot}^{\lambda_{irr}}\left(t\right)}\;P_{0}^{\lambda_{irr}}\;\left(1-10^{-\;A_{tot}^{\lambda_{irr}}\left(t\right)}\right)=A_{Y_{j\;or\;j^{\prime }}}^{\lambda_{irr}}\left(t\right)\;P_{0}^{\lambda_{irr}}\;PKF^{\lambda_{irr}}\left(t\right),$$ (2)

with the dimensionless total absorbance ( $A_{tot}^{\lambda_{irr}}\left(t\right)$ , Eq. (3)) being a sum of the individual absorbances of the $n_{sp}$ species present at time t and at $\lambda_{irr}$ ( $A_{Y_{j}}^{\lambda_{irr}}\left(t\right)$ ).

$$A_{tot}^{\lambda_{irr}}\left(t\right)=\sum_{j=0}^{n_{sp}}A_{Y_{j}}^{\lambda_{irr}}\left(t\right)=\sum_{j=0}^{n_{sp}}\varepsilon_{Y_{j}}^{\lambda_{irr}}\;l_{irr}C_{Y_{j}}^{Lp,∆\lambda }\left(t\right).$$ (3)

In Eq. (2), $P_{0}^{\lambda_{irr}}$ is the incident number of photons at $\lambda_{irr}$ that enter the reactor per second and per irradiated area and volume of the investigated sample, expressed in $einstein\;s^{-1}\;{dm}^{-3}$ (Maafi, 2023). $PKF\left(t\right)$ is the dimensionless photokinetic factor. $\varepsilon_{Y_{j}}^{\lambda_{irr}}$ (in ${M^{-1}\;cm}^{-1}$ , Eq. (3)), is the absorptivity of species $Y_{j}$ at $\lambda_{irr}$ , and $l_{irr}$ (in cm ), the optical path length of the irradiation light inside the sample.

The dimensions of the right- ( $einstein\;s^{-1}\;{dm}^{-3}$ ) and left- ( $mol\;{dm}^{-3}\;s^{-1}$ ) hand sides of Eq. (1) are equivalent because an einstein is equal to the Avogadro number of photons.

The quantum yield (Eq. (1)), as well as all the parameters in Eq. (2), are considered here to be wavelength-dependent. In addition, the rate and the concentration in Eq. (1) are labeled by both the wavelength range of the *OSIA* ( ∆λ ) and the particular lamp used ( Lp ). The justification for such labeling is provided in Section 3.9.

It is interesting to observe that the right-hand side of Eq. (1) accounts only for the non-zero ( $\Phi_{Y_{j}\;\to \;Y_{j^{\prime }}}^{\lambda_{irr}}$ ; $\Phi_{Y_{j^{\prime }}\;\to Y_{j}}^{\lambda_{irr}}$ ; $P_{0}^{\lambda_{irr}}$ ; $\varepsilon_{Y_{j}}^{\lambda_{irr}}$ ) parameters. This means that Eq. (1) faithfully translates the impact of those parameters that effectively induce a change in the rate.

Unfortunately, solving Eq. (1) analytically is impossible except for very scarce (mostly hypothetical) cases that impose particular conditions on the shapes of the mathematical functions that map out the variations of the parameters of Eq. (1) with wavelength. In contrast to some well-known formulations of integro-differential equations that can be analytically solved (Brunner, 2017; Lewis et al., 2022), the type of Eq. (1) has not, as far as we are aware, benefited from even a standard numerical integration method able to evaluate such an equation. This might be, at least in part, due to the fact that the explicit formulae giving the parameters under the integral as functions of the wavelength ( $\Phi_{Y_{j}\;\to \;Y_{j^{\prime }}}^{\lambda_{irr}}$ ; $\Phi_{Y_{j^{\prime }}\;\to Y_{j}}^{\lambda_{irr}}$ ; $P_{0}^{\lambda_{irr}}$ ; $\varepsilon_{Y_{j}}^{\lambda_{irr}}$ ) are generally not known, and these formulae vary from one reaction case to another (no general expressions can fit all situations).

One way to circumvent this hurdle and achieve numerical integration is to replace the integration over the wavelength by a summation with a 1-nm-interval step (Eq. (4)). Such an approximation, using the Euler numerical method, has proved satisfactory to replicate experimental data (Lente and Espenson, 2004; Fabian and Lente, 2010). Accordingly, a fourth-order Runge–Kutta method has been implemented in the subsequent sections for the evaluation of the integro-differential equation (Eq. (4)) in various reaction conditions.

$$r_{Y_{j}}^{Lp,∆\lambda }\left(t\right)=\frac{dC_{Y_{j}}^{Lp,∆\lambda }\left(t\right)}{dt}=\sum_{\lambda_{irr}=\lambda_{a}}^{\lambda_{b}}\left(\sum_{j^{\prime }\;;\;j^{\prime }\neq j}^{n_{\Phi j}}\left(-\;\Phi_{Y_{j}\;\to \;Y_{j^{\prime }}}^{\lambda_{irr}}\;P_{a_{Y_{j}}}^{\lambda_{irr}}\left(t\right)+\Phi_{Y_{j^{\prime }}\;\to Y_{j}}^{\lambda_{irr}}\;P_{a_{Y_{j^{\prime }}}}^{\lambda_{irr}}\left(t\right)\right)\right)=\sum_{\lambda_{irr}=\lambda_{a}}^{\lambda_{b}}r_{Y_{j}}^{\lambda_{irr}}\left(t\right).$$ (4)

The initial reactant rate ( $r_{\;Y_{0}}^{Lp,∆\lambda }\left(0\right)=r_{X}^{Lp,∆\lambda }\left(0\right)=r_{0,X}^{Lp,∆\lambda }$ , Eq. (5)) is extracted from Eq. (4). It represents the highest value of the reactant rate at all times ( $r_{0,X}^{Lp,∆\lambda }\geq r_{X}^{Lp,∆\lambda }\left(t\right)$ ).

$$r_{X}^{Lp,∆\lambda }\left(0\right)=\sum_{\lambda_{irr}=\lambda_{a}}^{\lambda_{b}}r_{X}^{\lambda_{irr}}\left(0\right)=r_{0,X}^{Lp,∆\lambda }=-\sum_{\lambda_{irr}=\lambda_{a}}^{\lambda_{b}}\left(\sum_{j^{\prime }\;;\;j^{\prime }\neq 0}^{n_{\Phi_{0}}}\left(\Phi_{X\;\to \;Y_{j^{\prime }}}^{\lambda_{irr}}\;P_{0}^{\lambda_{irr}}\;\left(1-10^{-\;A_{X}^{\lambda_{irr}}\left(0\right)}\right)\right)\right).$$ (5)

### 3.2 The global integrated rate law model

As discussed above, it is a matter of fact that the literature provides neither an explicit equation for the kinetics traces of photoreactions driven by polychromatic light nor an analytical solution to the integro-differential rate equation (Eq. (1)). However, a qualitative observation of the overall shape of experimental and RK-calculated traces of such photoreactions indicates that they do not seem to substantially differ from those recorded for reactions subjected to monochromatic light (Lente and Espenson, 2004; Fabian and Lente, 2010; Roibu et al., 2018; Volfova et al., 2019; Yutani et al., 2019; Maafi and Al Qarni, 2022a; Maafi and Al Qarni, 2022b; Weingartz et al., 2023). This observation allows one to conjecture that the kinetics under polychromatic light may well be of Φ -order character, similar to that previously established for the photokinetics of systems exposed to monochromatic light (Maafi, 2023). If this suggestion is valid, the traces would be characterized by the same global equation established earlier for systems exposed to monochromatic light. Eq. (6) is then valid for any species $Y_{j}$ of any photoreaction, driven by polychromatic light, irrespective of the photomechanism in play.

$$C_{Y_{j}}^{Lp,∆\lambda }\left(t\right)=C_{\infty ,j}^{Lp,∆\lambda }+\sum_{i=1}^{i_{j}}\omega_{ij}^{∆\lambda }\;\mathrm{Log}\left(1+{cc}_{j}^{∆\lambda }e^{-\;k_{ij}^{∆\lambda }\;t}\right).$$ (6)

The parameters in Eq. (6) mirror those previously defined for the monochromatic light equation, except that they are now defined for the *OSIA* wavelength range, ∆λ . These parameter values are worked out from the fitting of Eq. 6 to the species $Y_{j}$ trace. The number ( $i_{j}$ ) of mono- Φ -order terms ( $\omega \;\mathrm{Log}\left(1+cc\;e^{-\;k\;t}\right)$ ) in the sum of Eq. (6) is equal to the number ( $n_{\Phi j}$ ) of photochemical reaction steps starting or ending at the considered species $Y_{j}$ ( $i_{j}\leq n_{\Phi_{j}}$ ), except for cyclic reactions where $i_{j}\leq n_{\Phi }$ . The *log-exp* format of Eq. (6) embodies its Φ -order character, with Log being the base 10 logarithm and e the exponential function.

The differentiation of Eq. (6) yields the general expression of the rate of $Y_{j}$ reaction at time t (Eq. (7)).

$${\left(C_{Y_{j}}^{Lp,∆\lambda }\left(t\right)\right)}^{\prime }=r_{Y_{j}}^{Lp,∆\lambda }\left(t\right)=-\sum_{i=1}^{i_{j}}\frac{\omega_{ij}^{∆\lambda }\;{cc}_{j}^{∆\lambda }\;k_{ij}^{∆\lambda }\;e^{-\;k_{ij}^{∆\lambda }\;t}}{\left(1+{cc}_{j}^{∆\lambda }\;e^{-\;k_{ij}^{∆\lambda }\;t}\right)\;\ln\left(10\right)}.$$ (7)

The initial reaction rate (Eq. (8)) is derived from Eq. (7) and serves as a metric to quantify the reactivity of $Y_{j}$ .

$${\left(C_{Y_{j}}^{Lp,∆\lambda }\left(t\right)\right)}_{t=0}^{\prime }={Fit:r}_{0,Y_{j}}^{Lp,∆\lambda }=-\sum_{i=1}^{i_{j}}\frac{\omega_{ij}^{∆\lambda }\;{cc}_{j}^{∆\lambda }\;k_{ij}^{∆\lambda }\;}{\left(1+{cc}_{j}^{∆\lambda }\right)\;\ln\left(10\right)}.$$ (8)

Similarly, the general equation describing a total absorption of the medium measured at both a given observation wavelength $\lambda_{obs}$ and an observation optical path length $l_{obs}$ is given by Eq. (9). Both $\lambda_{obs}$ and $l_{obs}$ might or might not be equal to their irradiation counterparts ( $\lambda_{irr}$ and $l_{irr}$ ). The number, $i_{A}$ , of mono- Φ -order terms in Eq. (9) cannot exceed the number ( $n_{\Phi }$ ) of photochemical reaction steps involved in the reaction mechanism ( $i_{A}\leq n_{\Phi }$ ) but can be less than that number depending on the shape of the trace.

$$A_{tot}^{∆\lambda /\lambda_{obs}}\left(t\right)=A_{tot}^{∆\lambda /\lambda_{obs}}\left(\infty \right)+\sum_{i=1}^{i_{A}}\omega_{i,A}^{∆\lambda }\;\mathrm{Log}\left(1+{cc}_{A}^{∆\lambda }e^{-\;k_{iA}^{∆\lambda }\;t}\right),$$ (9)

with a general initial rate for the total absorbance trace being

$${\left(A_{tot}^{∆\lambda /\lambda_{obs}}\left(t\right)\right)}_{t=0}^{\prime }={Fit:r}_{0,A}^{Lp,∆\lambda }=-\sum_{i=1}^{i_{A}}\frac{\omega_{i,A}^{∆\lambda }\;{cc}_{A}^{∆\lambda }\;k_{iA}^{∆\lambda }}{\left(1+{cc}_{A}^{∆\lambda }\right)\;\ln\left(10\right)}.$$ (10)

### 3.3 Specification of reaction intrinsic parameters and incident light

Prior to verifying the validity of the global equation (Eq. (6)) for the description of the output RK-data of reaction photokinetics (see Sections 3.4–3.9), we ought to ask how to proceed with the determination of the values of essential quantities required for RK-calculations at each $\lambda_{irr}$ of the irradiation range, that is, quantum yields, absorptivities of the species, radiation intensities, and the actual reaction photomechanism. For the latter, it is preferable that the experimentalist knows the reaction mechanism in play before engaging in the analysis of the photokinetic data of the reaction (even though, as we shall see, this is not imperative).

The unique way to determine the values of the intrinsic parameters ( $\Phi_{Y_{j}\;\to Y_{j^{\prime }}}^{\lambda_{irr}}$ , $\Phi_{Y_{j^{\prime }}\;\to Y_{j}}^{\lambda_{irr}}$ , and $\varepsilon_{Y_{j}}^{\lambda_{irr}}$ ) experimentally is by conducting an investigation whereby the reactive medium is successively exposed to individual monochromatic lights belonging to the irradiation range, ∆λ . The kinetic data collected accordingly, are analyzed by the methods and procedures described previously (Maafi, 2023) to solve for these intrinsic photoreaction parameters. Only a physical actinometer can determine the radiation intensities, $P_{0}^{\lambda_{irr}}$ , that is, the emission profile of the lamp (e.g., a spectroradiometer). Therefore, if these quantities are required, they must be acquired separately and before the polychromatic study is performed, since the latter cannot deliver such quantities. Such values are considered known when the RK calculation is performed. The generated RK trace data from simulated situations allow for exploring much of what photokinetics under polychromatic light can deliver. This is discussed in the following sections.

### 3.4 Validation of the global equation

More than 250 kinetic traces were RK-calculated for various reactions and reaction conditions. They are considered a representative sample of photoreactions exposed to polychromatic light. The selected reactions differed by the photomechanisms, the intrinsic features (variation over the wavelength of both species quantum yield patterns and values and absorption coefficient for each species), and the experimental conditions, including the lamp profile/intensity and the irradiation and observation optical path lengths. These traces, as well as those corresponding to the total absorption of the reaction medium, were fitted by Eqs. (6)–(9) of adequate numbers $i_{j}$ and $i_{A}$ , respectively.

**FIGURE 2:**
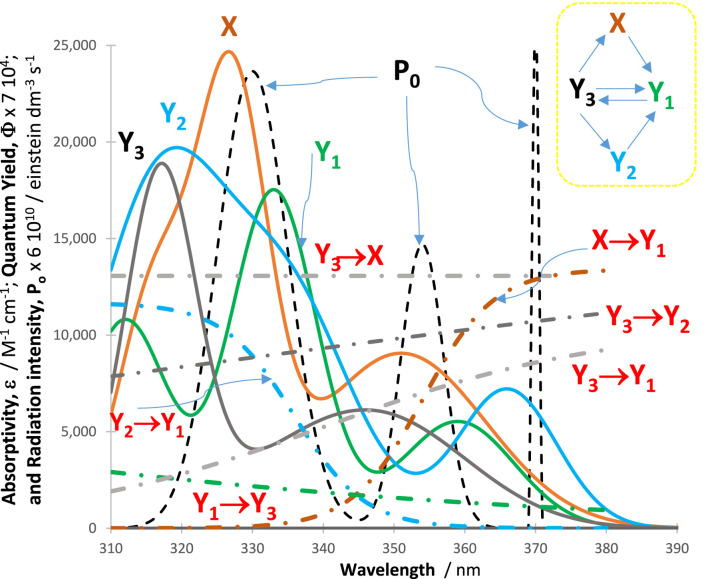
Intrinsic parameters $\Phi_{Y_{j}\;\to Y_{j^{\prime }}}^{\lambda_{irr}}$ and $\Phi_{Y_{j^{\prime }}\;\to Y_{j}}^{\lambda_{irr}}$ (dash-dotted lines) and $\varepsilon_{Y_{j}}^{\lambda_{irr}}$ (solid lines) of the cyclic reaction indicated in the top right corner and the lamp profile ( $P_{0}^{\lambda_{irr}}$ , black dashed line) used for irradiation of the reactive medium.

**FIGURE 3:**
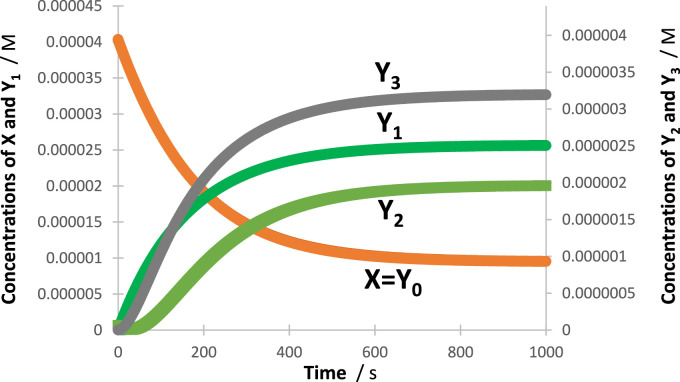
An illustration of the excellent fit of the RK-calculated traces (circles) of the tetramolecular cyclic reaction (shown in the inset of Figure 2) by the corresponding Eq. 6 (lines).

In all cases, an excellent fit of the traces by the model equations was found. High (>0.999) correlation coefficient values were found for RK-simulated vs. Eqs. (6)–(9) data, and relatively low values were found for the sums of squared errors (SSE <10^−10^) and root-mean-square errors (RMSE <10^−9^ for Eq. (6) and slightly higher, or 10^−5^, for Eq. (9) characterized the fit of each trace. Figure 2 shows an example of the profiles of quantum yields, absorptivities, and a lamp. The photomechanism corresponds to a cyclic reaction involving four species interlinked by six photoreaction steps. The traces of the species are fitted by Eq. (6) (Figure 3), each involving two mono- Φ -order terms ( $i_{j}=2$ ), with $k_{10}^{∆\lambda }=0.01654\;s^{-1}$ , and $k_{20}^{∆\lambda }=0.006357\;s^{-1}$ for all.

These findings indicate that the Φ -order character of the photokinetics is preserved when polychromatic light is driving the photoreaction. This leads to drawing a general conclusion stating that both mono- and polychromatic lights induce photoreactions to follow an overall Φ -order kinetic behavior. In addition, these findings validate the universal character of the model equation, Eq. (6), that describes the photokinetic traces obtained under polychromatic light. Eq. (6) is, therefore, the first example in the photochemistry literature of a general, explicit model equation of photochemical reactions obeying any photomechanism under polychromatic light irradiation, irrespective of the profile of the employed lamp (or light source). Looking forward, the latter statement might make a basis for a conjecture that would extend the validity of these equations to include the description of photoreaction kinetics irrespective of the reactor geometry and/or the spatial distribution of the incident light on the sample, as usually employed in engineering and real-life setups. It is also important to note that the treatment of the data of photokinetics under polychromatic light must not be performed by the fundamental equations describing a reaction under monochromatic light on the premise that its kinetic trace obeys Eq. (6) or behaves in the same manner overall.

From a practical viewpoint, it is relevant to mention that, on the one hand, using Eq. (6) (and Eq. (9)) does not necessarily require a priori knowledge of the specific photomechanism governing the reaction. On the other hand, using these equations will unavoidably lead to the occurrence of an identifiability issue. As previously discussed for reactions driven by monochromatic light irradiation (Maafi, 2023), the identifiability issue corresponds to the situation where different sets of fitting parameter values ( ω , cc , and k for Eq. (6) or (9)) might be obtained from different sets of initial values of these parameters wherefrom the fitting process starts. The number of these sets might not be infinite but is still high enough not to be ignored. Each parameter set, introduced in the corresponding Eq. (6) (or Eq. (9)), would convey an excellent fit of the considered kinetic trace obtained under polychromatic light. Because of the diversity of sets of parameters for a given Eq. (6) (or Eq. (9)), the knowledge of the “*true*” set of kinetic parameters is impossible to single out. This means that one is certain that the form of Eq. (6) (or Eq. (9)) is correct, but the identification of the set of parameters that correspond to the physical system investigated is out of reach. As a consequence, the identifiability issue impedes the usage of the rate constant ( k ) for the quantification of a species photoreactivity (it is not possible to identify the true rate constant of a given reaction step with certainty from Eq. (6) or Eq. (9)). Unfortunately, there are no currently available means to solve this identifiability problem.

However, for a given kinetic trace, the initial reaction rate remains a useful metric because its value does not change for the different fitting parameter sets that emerge from the identifiability issue (a similar situation was observed for monochromatic irradiation (Maafi, 2023)). In addition, the initial rate has the unique advantage of being worked out by three independent means: a) theoretically, ${Theo:r}_{0,Y_{j}}^{Lp,∆\lambda }$ (and ${Theo:r}_{0,A}^{Lp,∆\lambda }$ ), from the rate law Eq. (4) considered at t=0 , (but, incidentally, Eq. (4) does not provide an equation for k ); b) by numerical integration, ${RK:r}_{0,Y_{j}}^{Lp,∆\lambda }$ and ${RK:r}_{0,A}^{Lp,∆\lambda }$ , (whereas the rate constants are not accessible numerically); and c) from the fitting of the kinetic trace data at hand, ${Fit:r}_{0,Y_{j}}^{Lp,∆\lambda }$ and ${Fit:r}_{0,A}^{Lp,∆\lambda }$ , as given by Eq. (8)–(10), respectively. This introduces a mandatory condition, whereby it is necessary to confirm that $Fit:r_{0Y_{j}}^{Lp,∆\lambda }=Theo:r_{0Y_{j}}^{Lp,∆\lambda }=RK:r_{0Y_{j}}^{Lp,∆\lambda }$ , in all situations.

**FIGURE 4:**
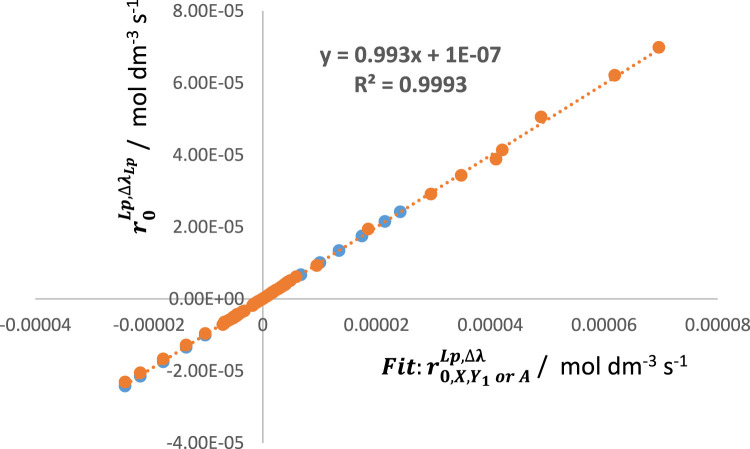
An excellent linear correlation between the values of $Fit:r_{0}^{Lp,∆\lambda }$ ( $Fit:r_{0,X}^{Lp,∆\lambda }$ , $Fit:r_{0,Y_{1}}^{Lp,∆\lambda }$ , and $Fit:r_{0,A}^{Lp,∆\lambda }$ ) against the respective $RK:r_{0}^{Lp,∆\lambda }$ and $Theo:r_{0}^{Lp,∆\lambda }$ ( $r_{0,A}^{Lp,∆\lambda }$ were scaled down by adequate multiplicative factors). Shown data belong to different reactive systems and experimental conditions (*n* > 220 data points).

In this context, linear relationships between, for instance, $Theo:r_{0Y_{j}}^{Lp,∆\lambda }$ and both $Fit:r_{0Y_{j}}^{Lp,∆\lambda }$ and $RK:r_{0Y_{j}}^{Lp,∆\lambda }$ are obtained for various reactive photosystems (Figure 4). This result attests to the reliability of the present methodology and further confirms the validity of the general model equation, Eq. (6).

### 3.5 An LED light is not monochromatic

It is now established that both polychromatic and monochromatic light equally induce Φ -order kinetics in photoreactions, which impedes the possibility of identifying which type of (mono- or polychromatic) light was employed to induce the reaction from the analysis of the traces’ shapes. Such a distinction can only be achieved from the instrumentation. Technically, a monochromatic light can only be produced by using a monochromator. Hence, an experimental setup that does not involve a monochromator produces a polychromatic beam (e.g., filtered lamp light). The case remains that LEDs are sometimes assumed to deliver monochromatic light (even if no monochromator is involved) (Stadler et al., 2018; Amouroux et al., 2019). This assumption is thought to facilitate the determination of the quantum yield of mainly the reactant by using the differential quantum yield equation (Braslavsky, 2007). In this respect, it is important to discuss whether the monochromaticity of LED light can be established/assumed.

Many examples of LED light profiles have been published (Volfova et al., 2019; Williams and Kappe, 2020). The data show that even if the mid-height width of the light bands produced by LEDs is much narrower than some of those recorded for classical lamps, it is, nonetheless, clear that their lights are far from being monochromatic. In addition, most blue LEDs are reported to emit a broad spectrum (Bonfield et al., 2020). Strictly speaking, an LED produces a polychromatic beam centered around a specific wavelength. In the visible region, this confers a particular uniform color to the emitted light that might mislead the observer to consider this light as monochromatic. Accordingly, the rate law for reactions performed under LED light exposure should take the form of the integro-differential equation (Eq. (1)). The latter is a sum of rates at individual wavelengths, which means that approximating the LED light to being monochromatic will certainly lead to errors. We shall see in Section 3.12 that there is little possibility of reliably evaluating the quantum yield when polychromatic light is employed. Hence, there is arguably no need for the monochromaticity of LEDs approximation. Incidentally, the quantum yield might well be wavelength-dependent even over the wavelength section covered by the relatively narrow light band of the LED (Maafi and Al Qarni, 2022a).

### 3.6. Spectator molecules’ impact on photoreactivity

In real-life situations, photoreactions may be performed in a medium that includes spectator molecules (*SPM*s) that do not directly contribute to the photoreaction investigated (they are both thermally and photochemically inert). Such molecules might be additives (excipient, dyes … etc.) fulfilling a purpose in the formulation or the reactive medium.

*SPM*s will have an impact on the photoreactivity of a reactive system driven by polychromatic light if they happen to absorb in the *OSIA* wavelength range of the species investigated. Such an absorption implies a necessary modification of the rate law of the reaction. The contribution of spectator molecules is mathematically expressed by including the absorption of *SPM*s into the total absorption of the reactive medium. This follows from the fact that *SPM*s, which are both thermally and photochemically unreactive, only impact the kinetics by competing for the available light over the *OSIA*. Hence, at every wavelength, $\lambda_{irr}$ , the total medium absorbance (Eq. (11)) accumulates the individual time-variable absorbances of reaction species as well as the constant absorbance of *SPM*s ( 0≤r≤w ).

$$A_{tot}^{\lambda_{irr}}\left(t\right)=\sum_{r=1}^{w}A_{{SPM}_{r}}^{\lambda_{irr}}+\sum_{j=0}^{n_{sp}}A_{Y_{j}}^{\lambda_{irr}}\left(t\right).$$ (11)

**FIGURE 5:**
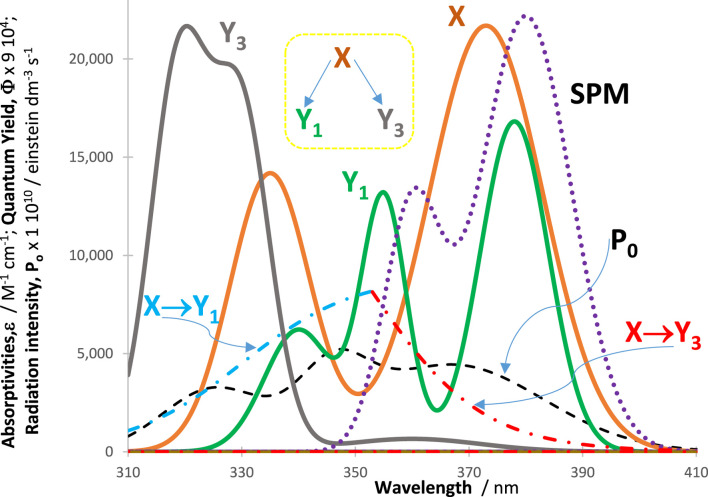
Absorptivities ( ε , solid lines) and quantum yields ( Φ , dash-dotted lines) of the divergent reaction’s species, the incident light profile ( $P_{0}$ , dashed line), and an example of the absorptivity envelope of the spectator molecules (*SPM*, dotted purple line) present in the reactive medium (the *SPM* concentration takes various values).

**FIGURE 6:**
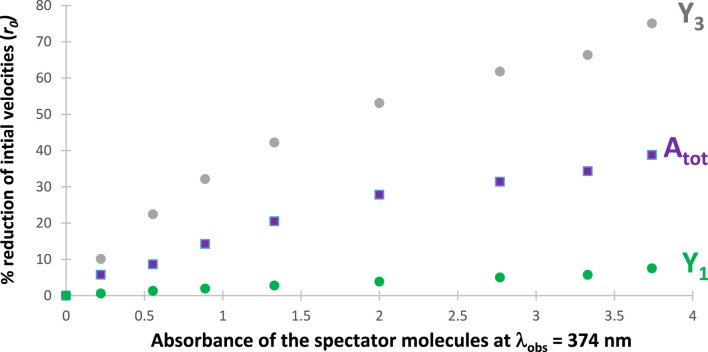
Percentage reduction of the initial speeds of both reaction products $Y_{1}$ and $Y_{3}$ , as well as that of the total absorbance of the medium, $A_{tot}$ , observed at 380 nm, with increasing absorbance of the spectator molecules measured at 374 nm. The values corresponding to the purple squares represent the initial rate of the total absorbance trace multiplied by a scaling factor $\left(-\;10^{-5}\;r_{o,A}\right)$ .

As a consequence, for a given initial concentration of the reactant, the higher the *SPM* absorption over ∆λ , the lower the $PKF\left(t\right)$ at the wavelengths concerned, which causes lower individual rates at these wavelengths, eventually leading to a lower global rate of the photoreaction (slowing of all species reactivities). An illustration of this phenomenon is depicted in Figure 5 and Figure 6. In this case, the reaction is a divergent depletion of the reactant ( $Y_{1}\leftarrow X\to \;Y_{3}$ ) by branched processes assuming, for instance, the reactivity of two separate but simultaneously irradiated functional groups within a single molecule. Here, it is considered that the *SPM* absorption covers only the absorption band of one of the functional groups but not the other (Figure 5), which is a typical situation exploited for chromatic orthogonality (Bochet, 2006; Corrigan and Boyer, 2019; Kumar et al., 2023). For our example, the increase of the selected *SPM*s’ absorption blocks one reaction step almost completely (e.g., that producing $Y_{3}$ , corresponding to the reactant absorption band situated between 350 and 410 nm), which allows photoselectivity of the product ( $Y_{1}$ ) emerging from the other reaction branch, which corresponds to the reactant absorption band situated between 310 and 350 nm (or vice versa for another set of *SPM*s whose absorption region overlaps that of $Y_{1}$ ).

It is remarkable that the initial rate of $Y_{3}$ reduces by *ca*. 80% compared to its value in the absence of *SPM*s, whereas that of $Y_{1}$ records less than 10% decrease (Figure 6).

The reduction of photoreactivity by introducing *SPM*s in the medium was also confirmed for many RK-simulated reactive photosystems under polychromatic light irradiation. This behavior is an expansion to multiwavelength irradiation of a similar effect of *SPM*s that has been confirmed by RK calculations and experimentally for photoreactions exposed to monochromatic light (Maafi, 2023). The quantification for our example can proceed by monitoring the rate of decrease of the initial products ( $Y_{1}$ and $Y_{2}$ ) or by the initial rates worked out from the reactant concentration or the total absorbance traces (the latter are measured at a given observation wavelength with increasing *SPM* absorption). An example is shown in Figure 6.

### 3.7 Auto-photostabilization due to initial reactant concentration

Except for photoreactions, where the reactant is the only absorbing species in the reactive medium, changing the magnitude of the initial concentration of the reactant, $C_{X}\left(0\right)$ (with all remaining reaction attributes being the same) results in a measurable impact on the photoreactivity of Φ -order reactions. It has been previously demonstrated that photoreactions under monochromatic light gradually slowdown for higher values of $C_{X}\left(0\right)$ (Maafi, 2023). The interpretation of this pattern follows from the fact that an increase of $C_{X}\left(0\right)$ causes the increase of the term $\left(1-10^{-A_{tot}^{\lambda_{irr}}}\right)$ and, therefore, an increase of the absolute value of the initial reactant rate (Eq. (5)). However, the same effect leads to a reduction of the photokinetic factor ( $\left(1-10^{-A_{tot}^{\lambda_{irr}}}\right)/A_{tot}^{\lambda_{irr}}$ ), which causes a reduction of the overall rate and hence slows the reaction. This explanation holds for both reactant and photoproducts, irrespective of the photomechanism in play.

**FIGURE 7:**
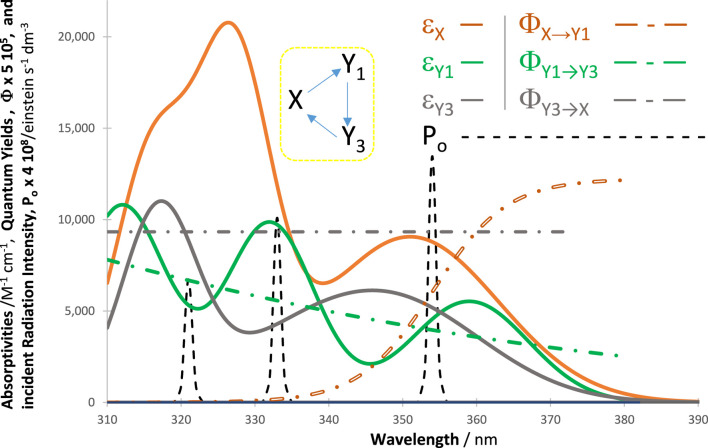
Properties ( ε and Φ ) of the tricyclic reaction (mechanism shown in the inset) and profile of the lamp’s polychromatic light to which the reactive system is subjected.

**FIGURE 8:**
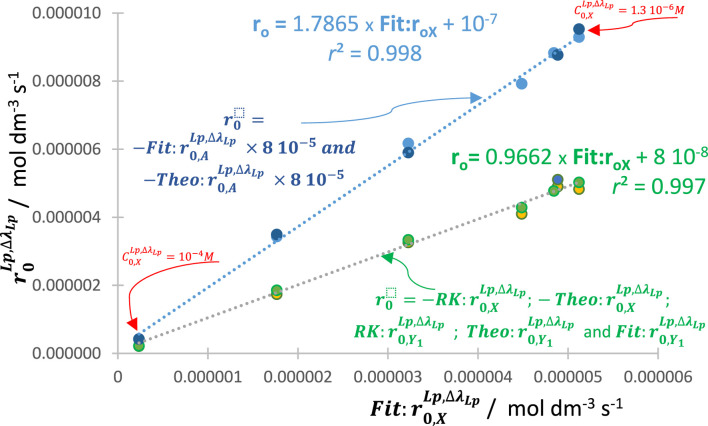
Variation of the initial rates of reactant (of the reaction shown in Figure 7), $RK:r_{0X}^{Lp,∆\lambda }$ and $Theo:r_{0X}^{Lp,∆\lambda }$ , photoproduct $Y_{1}$ , $Fit:r_{0Y_{1}}^{Lp,∆\lambda }$ , $RK:r_{0Y_{1}}^{Lp,∆\lambda }$ , and $Theo:r_{0Y_{1}}^{Lp,∆\lambda }$ , and that of the total absorbance trace, $Fit:r_{0A}^{Lp,∆\lambda }$ and $Theo:r_{0A}^{Lp,∆\lambda }$ , with the initial reactant rate $Fit:r_{0X}^{Lp,∆\lambda }$ obtained from the fitting of traces corresponding to initial reactant concentrations of $1.3\;10^{-6}$ , $1.3\;10^{-5}$ , $3\;10^{-5}$ , $5\;10^{-5}$ , $7\;10^{-5}$ , $9\;10^{-5}$ , and $10^{-4}\;M$ (all belong to the linearity range of the reactant calibration graph).

Conceptually, the above pattern is preserved when the radiation type changes from monochromatic to polychromatic light because, as seen in Section 3.4, the Φ -order character of the photokinetics is conserved (traces are fitted by Eq. (6)). Therefore, as predicted and irrespective of the mechanism of the photoreaction, the analysis of RK-simulated traces shows that $r_{0,X}^{Lp,∆\lambda }$ increased whereas the reaction slowed when the initial concentration values increased. An illustration of this behavior is provided for the trimolecular cyclic photoreaction subjected to a polychromatic beam (Figure 7), whose initial rates for reactant X and photoproduct $Y_{1}$ , as well as that worked out for the total absorbance measured at 326 nm, are reported in Figure 8.

Such effects prove the occurrence of an auto-photostabilization of the reaction by higher values of the initial concentration of the reactant when polychromatic radiation drives the reactive medium. Notice that this conclusion is only valid when the concentrations of the light-absorbing reactive species fall within their respective spectrophotometric linearity ranges of the calibration graphs (i.e., where Eq. (1) is consistent and is the basis for the RK numerical calculations).

In analogy with the monochromatic irradiation results (Maafi, 2023), constant ratios, invariant with $C_{X}^{Lp,∆\lambda }\left(0\right)$ values, have also been recorded for the final concentrations of pairs of species that either reach photostationary states or are end products when polychromatic light is used.

A similar decrease/increase of the reaction rate to that shown above with higher/lower values of $C_{X}^{Lp,∆\lambda }\left(0\right)$ occurs when high/small values of the irradiation optical path length ( $l_{irr}$ ) are considered. This follows from the concomitant presence of these two parameters as a product ( $C_{X}^{Lp,∆\lambda }\left(0\right)\times l_{irr}$ ) within the formula of the absorbance (Eq. (3)). Furthermore, varying one or the other of the latter parameters would cause an equivalent effect to proportionally changing the intrinsic parameter $\varepsilon^{\lambda_{irr}}$ over the irradiation range ∆λ to higher/lower values (also because of its presence in the absorbance formula). This corresponds to virtually investigating a series of species, such as derivatives of the same molecular system with different absorption coefficients. This feature could be exploited in optimizing a reactive system for a given application.

The auto-photostabilization phenomenon and its quantification is an additional proof for i) the validity of the general model equation, Eqs (6), ii) the usefulness of $r_{0,X}^{Lp,∆\lambda }$ as a metric, and iii) the efficiency of RK calculations to investigate and quantify the photokinetic behavior of photoreactive systems.

The correlation of $r_{0,X}^{Lp,∆\lambda }$ with $r_{0,A}^{Lp,∆\lambda }$ (Figure 8) comes from the proportionality between the variation of initial velocities of the reactant and photoproduct $\left(r_{0X}^{Lp,∆\lambda }/r_{0Y_{1}}^{Lp,∆\lambda }=constant\right)$ that confers a proportionality of $r_{0X}^{Lp,∆\lambda_{Lp}}$ and $r_{0A}^{Lp,∆\lambda_{Lp}}$ because the latter is a linear combination of $r_{0X}^{Lp,∆\lambda_{Lp}}$ and $r_{0Y_{1}}^{Lp,∆\lambda_{Lp}}$ . This should hold irrespective of the photomechanism governing the reaction and/or the experimental conditions imposed on the reactive system. Incidentally, the reactivity of all photoproducts is somewhat proportional to the variation of $r_{0X}^{Lp,∆\lambda }$ in the same direction (any increase of $r_{0X}^{Lp,∆\lambda }$ is followed by a proportional increase of reactivity of all subsequent photoproducts, and vice versa).

### 3.8 Reactivity causative wavelength region

The polychromaticity of the lamp over a wavelength range ∆λ brings up the question of whether there is a section ( $∆\lambda_{WROR}$ ) within the *OSIA* ( ∆λ ) that can induce a relatively higher reactivity (i.e., highest values for the rate) of the investigated system than other wavelength sections within ∆λ . This particular section is dubbed here the wavelength range of optimal reactivity (*WROR)*. Unfortunately, the literature fails to provide a means by which to address this matter and identify *WROR*. Here, we explore rate-law-based tools for that purpose.

In the case of the Φ -shaped reaction mechanism (Maafi, 2023), the initial reactant rate is derived from Eq. (4) for $Y_{3}\leftarrow X\;\to \;Y_{1}$ , as

$$r_{X}^{Lp,∆\lambda }\left(0\right)=r_{0,X}^{Lp,∆\lambda }=-\;\left(C_{X}^{Lp,∆\lambda }\left(0\right)\;l_{irr}\right)\sum_{\lambda_{irr}=\lambda_{a}}^{\lambda_{b}}\left(\left(\Phi_{X\;\to \;Y_{1}}^{\lambda_{irr}}+\Phi_{X\;\to \;Y_{3}}^{\lambda_{irr}}\right)\;\varepsilon_{X}^{\lambda_{irr}}\;P_{0}^{\lambda_{irr}}\;PKF^{\lambda_{irr}}\left(0\right)\;\right)=\sum_{\lambda_{irr}=\lambda_{a}}^{\lambda_{b}}r_{0,X}^{\lambda_{irr}}.$$ (12)

For given reaction conditions, the value of $r_{0,X}^{Lp,∆\lambda }$ is unique, encompassing the features of the reaction over the whole ∆λ domain, as expressed by Eq. (12). Therefore, this global equation cannot provide details on distinct segments within ∆λ , (e.g., $r_{0,X}^{Lp,∆\lambda }=r_{0,X}^{Lp,∆\lambda_{WROR}}+r_{0,X}^{Lp,∆\lambda_{1}}+r_{0,X}^{Lp,∆\lambda_{2}}+\ldots$ , with $∆\lambda =∆\lambda_{WROR}+∆\lambda_{1}+∆\lambda_{2}+\ldots$ ).

Nonetheless, notice that the last term of Eq. (12) indicates that individual wavelength $r_{0,X}^{\lambda_{irr}}$ values must vary over the domain ∆λ in proportion to the magnitude of the reaction features and parameters at each wavelength. The profile of $r_{0,X}^{\lambda_{irr}}$ over ∆λ is most likely not to be monotonical but probably non-linear and curved. This means that the $r_{0,X}^{\lambda_{irr}}$ profile should eventually present maxima and minima. The highest maximum on the $r_{0,X}^{\lambda_{irr}}$ profile would define the centre of the *WROR* and, hence, identify/estimate $∆\lambda_{WROR}$ . A careful consideration of Eq. (12) reveals, however, that the profile of $r_{0,X}^{\lambda_{irr}}$ is a function of the profiles of the intrinsic parameters ( $\varepsilon_{X}^{\lambda_{irr}}$ and $\Phi_{X\;\to \;Y_{j}}^{\lambda_{irr}}$ ), and $P_{0}^{\lambda_{irr}}$ , for given values of initial concentration, $C_{X}^{Lp,∆\lambda }\left(0\right)$ , and irradiation optical path length, $l_{irr}$ . The intrinsic parameters are inherent to the investigated reactant and cannot be modified, but the $P_{0}^{\lambda_{irr}}$ -pattern is a property of the lamp used to irradiate the sample. The profile of $r_{0,X}^{\lambda_{irr}}$ is then necessarily dependent on the radiation profile, which is an interdependence that predicts a variability of the reactivity with the light source. For instance, lamps with different profiles might well lead to different *WROR*s for the same reactant X . To resolve such an issue, the *WROR* can be uniquely specified if we consider the hypothetical case where the same number of photons ( $P_{0}^{\lambda_{irr}}=P_{0}$ ) are delivered to the reaction medium at each wavelength over ∆λ . For this situation, the profile of $r_{0,X}^{\lambda_{irr}}$ becomes independent of $P_{0}$ (but its ( $r_{0,X}^{\lambda_{irr}}$ ) values will still depend on wavelength). Unfortunately, because a lamp, $Lp_{Hyp}$ , providing a constant $P_{0}^{\lambda_{irr}}$ , does not yet exist, it is, *de facto*, not possible to determine *WROR* experimentally using a single lamp. The way forward for the specification of *WROR* is then either through calculation or by using several lamps of narrow band (LEDs) and tuning their $P_{0}$ to be the same (by a fine-alteration of the electric power received by each LED).

Incidentally, the above discussion clearly shows that it is not accurate to claim that the rate of the reaction should be maximal at the maximum of either the absorption ( $\varepsilon_{X}^{\lambda_{irr}}$ ) of the reactant or that of the radiation intensity profile ( $P_{0}^{\lambda_{irr}}$ ). Eq. (12) shows that the profile of $r_{0,X}^{\lambda_{irr}}$ should follow that of the product, $\varepsilon_{X}^{\lambda_{irr}}\times \Phi_{X\;\to \;Y_{j}}^{\lambda_{irr}}$ (when $P_{0}^{\lambda_{irr}}=P_{0}$ ). This result stresses the importance of a careful determination of the intrinsic parameters $\varepsilon_{X}^{\lambda_{irr}}$ and $\Phi_{X\;\to \;Y_{j}}^{\lambda_{irr}}$ (when $P_{0}^{\lambda_{irr}}$ , $C_{X}^{Lp,∆\lambda }\left(0\right)$ , and $l_{irr}$ all have constant values). (This aspect was facilitated by the methods provided in a previous study (Maafi, 2023)).

Hence, it is predicted that the patterns of $r_{0,X}^{\lambda_{irr}}$ are similar to those of $\left(\varepsilon_{X}^{\lambda_{irr}}\times \Phi_{X\;\to \;Y_{j}}^{\lambda_{irr}}\right)$ . The highest region on both patterns defines *WROR*.

**SCHEME 2:**
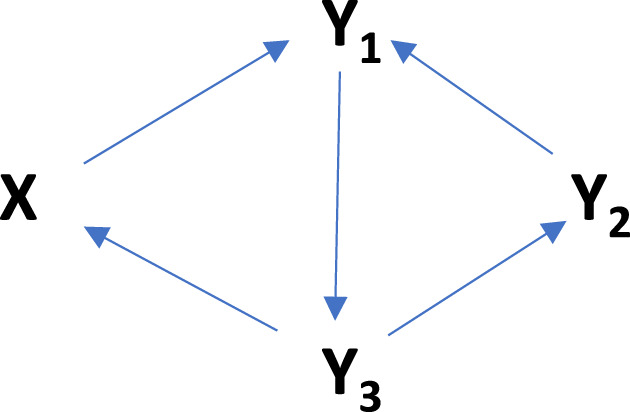
The tetramolecular cyclic reaction used to illustrate the evaluation of the WROR region.

**FIGURE 9:**
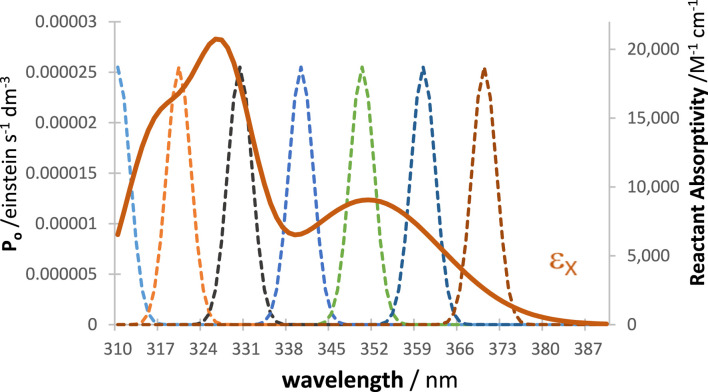
The absorption coefficient of the reactant ( $\varepsilon_{X}$ , plain line) and the profiles of a series of LED-like radiations whose maxima lay between 310 nm and 370 nm (dashed lines), each delivering the same number of photons to the medium.

In order to prove this point, we consider, for illustration, a tetramolecular cyclic reaction (Scheme 2), successively under irradiation by a series of h LED-like lamps ( 1≤h≤7 ) of relatively narrow emission bands, delivering the same number of photons to the reactor, whose maxima cover the whole absorption spectrum of the reactant (Figure 9).

**FIGURE 10:**
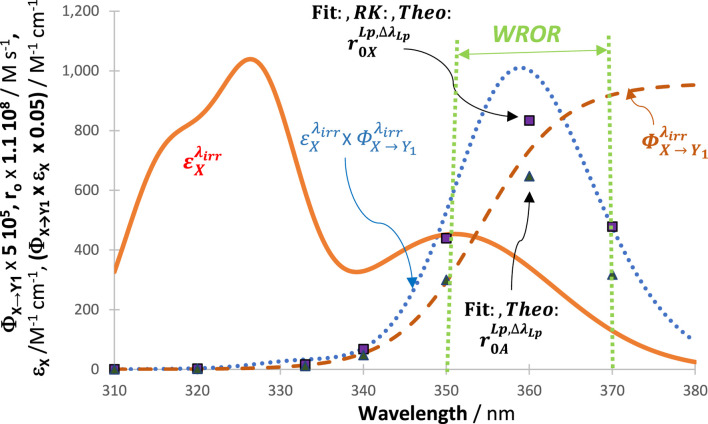
Variation of reactivity of the system (measured by the negative values of $Fit:,Theo:and\;RK:r_{0,X}^{Lp_{h},∆\lambda_{h}}$ or $Fit:,Theo:and\;RK:r_{0,Y_{1}}^{Lp_{h},∆\lambda_{h}}$ (plain squares) and $Fit:and\;Theo:r_{0,A}^{Lp_{h},∆\lambda_{h}}$ (plain triangles)), induced by the series of LED-like irradiation bands (Figure 9). The reactant absorption spectrum (solid line), the reactant quantum yield (dashed line), and the profile of the product $\left(\varepsilon_{X}^{\lambda_{irr}}\times \Phi_{X\;\to \;Y_{j}}^{\lambda_{irr}}\right)$ (dotted line) are provided for comparison.

RK traces of the reactive species and of the total absorbance at 325 nm were calculated for every LED shown in Figure 9, and each trace was fitted with an Eq. 6 involving four mono- Φ -order terms. The initial reactant rates ( $r_{0,X}^{Lp_{h},∆\lambda_{h}}$ , $r_{0,Y_{1}}^{Lp_{h},∆\lambda_{h}}$ and $r_{0,A}^{Lp_{h},∆\lambda_{h}}$ ) obtained from these irradiations had excellent correspondence between the values generated theoretically, RK-calculated, and obtained from the fittings with Eq. (6). These values of $r_{0}^{Lp_{h},∆\lambda_{h}}$ were also individually plotted for comparison with the profile of the plot of the product $\left(\varepsilon_{X}^{\lambda_{irr}}\times \Phi_{X\;\to \;Y_{j}}^{\lambda_{irr}}\right)$ . Figure 10 clearly indicates that the patterns of the latter product and those of $r_{0}^{Lp_{h},∆\lambda_{h}}$ are similar, displaying a clear maximal value close to 360 nm. This value also defines the maximum of *WROR.* The highest reactivity of the studied system occurs when irradiation is performed at $∆\lambda_{WROR}=350-370\;nm$ . These findings confirm that the maximum of *WROR* does not necessarily coincide with an absorption maximum of the reactant (only when the quantum yield is constant will the *WROR* match the maximum absorption spectrum). Practically, the *WROR* can be defined by plotting the product $\left(\varepsilon_{X}^{\lambda_{irr}}\times \Phi_{X\;\to \;Y_{j}}^{\lambda_{irr}}\right)$ with no need of the individual values of $P_{0}^{\lambda_{irr}}$ at every wavelength (which are, often, not accessible, even if the general profile of the lamp might be available from the manufacturer of that lamp). Incidentally, the variation observed in the kinetics of a photosystem due to a change of the *OSIA* justifies the labeling of the concentration and other kinetic features in the general Eq. (4), with the wavelength range ∆λ .

In Sections 3.6 and 7, it was shown that, on the one hand, the highest relative values attained by the rate of the reactant, among those recorded during reaction time, are precisely the values of $r_{0,X}^{Lp,∆\lambda }$ measured at t=0 , and on the other hand, the trend followed by the initial reactant rate ( $r_{0,X}^{Lp,∆\lambda }$ ) is effectively followed by the remaining species of the reaction considered ( $r_{0,X}^{Lp,∆\lambda }\;∼\;r_{0,Y_{j}}^{Lp,∆\lambda }$ ). For instance, an increase of $r_{0,X}^{Lp,∆\lambda }$ , due to a given change in the reaction conditions (e.g., different lamps) would mean a proportional increase in the production rates of the end products. These criteria underline the benefit of determining *WROR:* to help select the adequate lamp or LED to achieve maximal reactivity, which is valuable information to improve the cost-effectiveness of a technological process. Conversely, irradiation at a region far from *WROR* helps avoid or at least minimize degradation of the reactant, which is a relevant aspect for many applications, such as pharmaceutical drugs. The importance of defining the optimum wavelength of reactivity (*WROR*) has recently been discussed in relation to efficiency, safety, and economic issues for various materials (Walden et al., 2023).

**FIGURE 11:**
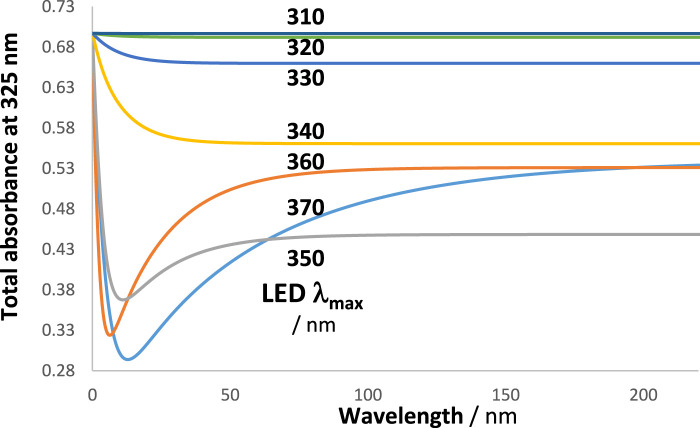
The kinetic trace pattern changes with the LED used. The traces correspond to the total absorbance measured at 325 nm (Figure 9). The maxima of the LED lights are indicated.

It is also interesting to look at the shape of the kinetic traces of the total absorbance when the maximum of the LED-like lamps (Figure 9) is gradually displaced within the absorption region of the investigated reaction (Scheme 2) while keeping the observation wavelength the same at 325 nm. In the case of our cyclic reaction, it turns out that $A_{tot}^{∆\lambda /\lambda_{obs}}\left(t\right)$ traces significantly differ from one another for different lamps (Figure 11).

As a general statement, the shape of the kinetic trace of a reaction is lamp-dependent, and the trace of its total absorbance is dependent on the observation wavelength. This corroborates a similar conclusion reached in experiments with diarylethene, dacarbazine, and nifedipine (Maafi and Al Qarni, 2022a; Maafi and Al Qarni, 2022b).

These results underline potential risks associated with directly comparing the kinetic traces and other kinetic outputs of reactions without carefully specifying all the conditions of the experiment and those relative to the acquisition of the kinetic data. This is one possible explanation for the difficulties experienced in the field when comparing photokinetic results, especially those obtained in different laboratories.

It is also straightforward to infer, from the above discussion, that for a given lamp, the kinetics of different reactions and/or molecules will not be directly comparable due to differences in their respective intrinsic parameters and their $\left(\varepsilon_{X}^{\lambda_{irr}}\times \Phi_{X\;\to \;Y_{j}}^{\lambda_{irr}}\right)$ products. In other words, reaction 1 is faster than reaction 2 only for the conditions used, but the relative speed is not absolute. For technological applications, comparing the efficiency of two processes in their respective optimal conditions is recommended.

The above findings lead us to conclude that the *OSIA* is practically easy to determine but does not give a precise account of the spectral region where the absorption of photons translates into a photokinetic change. The best way to specify such a region is to consider the wavelength span where the product $\left(\varepsilon_{X}^{\lambda_{irr}}\times \Phi_{X\;\to \;Y_{j}}^{\lambda_{irr}}\right)$ is non-zero, that is, where the rate (Eq. (4)) is not equal to 0 (in Figure 10, irradiation of X between 310 and *ca*. 330 nm, where it absorbs, has no effect because $\Phi_{X\;\to \;Y_{j}}^{\lambda_{irr}}$ is almost zero over this region). Obviously, such a precision supposes performing an extra series of experiments, data treatments, and the requirement of special instrumentation to determine the quantum yield profile with irradiation wavelength. Despite the possible imprecision, we will keep using *OSIA* for practicality (i.e., when the profile of the lamp is not known at the level of $P_{0}^{\lambda_{irr}}$ -values).

### 3.9 Kinactinometry

Actinometry measures the incident light flux per area and volume of the sample exposed to the light beam. When chemical actinometry is achieved on the basis of a photokinetic method, it is called “kinatinometry” (Maafi, 2023). This is the case of using Eq. 6 to determine the total amount of photons delivered by lamp Lp over a wavelength span of ∆λ per second and per irradiated area and volume of the sample investigated ( $P_{0}^{Lp,∆\lambda }$ is counted at the inner surface of the reactor before absorption by the photoreactive system). $P_{0}^{Lp,∆\lambda }$ represents a cumulative sum of photons delivered at individual wavelengths belonging to the irradiation domain ∆λ , that is, $P_{0}^{Lp,∆\lambda }=\sum_{\lambda_{a}}^{\lambda_{b}}P_{0}^{\lambda_{irr}}$ . As such, the numerical value of $P_{0}^{Lp,∆\lambda }$ neither informs on the range ∆λ (if not indicated on the labeling $P_{0}^{Lp,∆\lambda }$ ) nor on the profile of the lamp over ∆λ . In addition, the range ∆λ in $P_{0}^{Lp,∆\lambda }$ should in principle correspond to the *OSIA* of the species investigated (e.g., $∆\lambda_{Act}$ or $∆\lambda_{sp}$ ). In any case, the terms of the sum under the integral in Eq. (1) take non-zero values only when $P_{0}^{\lambda_{irr}}$ , $\varepsilon^{\lambda_{irr}}$ , and $\Phi_{Y_{j}\;\to \;Y_{j^{\prime }}}^{\lambda_{irr}}$ have all non-zero values. Hence, Eq. (4) automatically and exclusively depicts the kinetics owing to the lamp/reaction *OSIA*.

Practically, we ought to consider a few situations that determine the amount of photons that are counted relative to the reaction use. This requires considering the light-source profile ( $∆\lambda_{Lp}$ ), the actinometer (*OSIA*_*act*_), and the reaction (*OSIA*_*react*_) absorption domains relative to the lamp. First, let us consider only the lamp and the reactive species, where physical actinometry is performed to determine the number of photons delivered by the lamp.

In principle, the emitted light-source photons (over $∆\lambda_{Lp}$ ) that cross the irradiated area all reach the reactor. Here, we end up with two situations with respect to the absorption domain of the reaction studied. In the first instance (*sit*_*1*_), $∆\lambda_{Lp}$ encompasses but is larger than the *OSIA*_*sp*_ (e.g., *OSIA*_*sp*_ = 315–360 nm for $\varepsilon_{2}$ -species in Figure 1, but the lamp profile spans the $∆\lambda_{Lp}=$ 310–390 nm region). In this case, an excess number of photons is delivered to the reactor, where the photons with wavelengths outside the *OSIA*_*sp*_ are counted but not used by the reaction. If $∆\lambda_{Lp}$ is smaller than or equal to *OSIA*_*sp*_ (e.g., the *OSIA*_*sp*_ = 315–360 nm for $\varepsilon_{2}$ -species in Figure 1, and assuming the lamp profile spans only the $∆\lambda_{Lp}=$ 310–340 nm region), all the counted photons serve the reaction (*sit*_*2*_).

When chemical actinometry is used, it is required to take into account the actinometer (*OSIA*_*act*_) and the reaction (*OSIA*_*sp*_) absorption domains relative to the light-source profile ( $∆\lambda_{Lp}$ ). We observe *sit*_*2*_ when the *OSIA*s of the actinometer and species are the same and are larger than the wavelength domain covered by the lamp ( $∆\lambda_{Lp}<OSIA_{act}=OSIA_{sp}$ ), and *sit*_*1*_ when the actinometer has a larger *OSIA* than the species but narrower than the wavelength span delivered by the lamp ( $∆\lambda_{Lp}\geq OSIA_{act}>OSIA_{sp}$ ). For example, in Figure 1, *OSIA*_*sp*_ = 310–340 nm as $\varepsilon_{1}$ -species, *OSIA*_*act*_ = 310–365 nm as $\varepsilon_{2}$ -species, and the lamp profile spans the $∆\lambda_{Lp}=$ 310–390 nm region. In addition to *sit*_*1*_ and *sit*_*2*_, there is a rather unrealistic situation, *sit*_*3*_, that can occur when chemical actinometry is used, that is, when the species *OSIA* is larger than that of the actinometer ( $∆\lambda_{Lp}\geq OSIA_{sp}>OSIA_{act}$ ), leading to the species absorbing more photons than those counted by the actinometer. For example, *OSIA*_*act*_ = 310–340 nm as $\varepsilon_{1}$ -species, *OSIA*_*sp*_ = 310–365 nm as $\varepsilon_{2}$ -species, and the lamp profile spans the $∆\lambda_{Lp}=$ 310–390 nm region, as shown in Figure 1.

Accordingly, the number of photons measured by chemical actinometry for *sit*_*1*_ and *sit*_*3*_ are not coherent with Eq. (1). This undeniably will introduce errors in the determination of any photokinetic feature (including but not limited to the evaluation of the “quantum yield” and any built actinometric methods).

In this context, using Eq. (6) ensures that only the true reaction/lamp *OSIA* is accounted for (i.e., photons outside such an *OSIA* do not contribute to the experimentally observed or RK-simulated traces), hence conforming with Eq. (1). Therefore, a kinactinometric methodology developed on the basis of Eq. (6) offers greater reliability than existing actinometric methods.

The RK-simulations of reactive systems under polychromatic irradiation of increasing magnitude, for all the above situations, have shown an acceleration of the reaction as predicted from Eqs (1), (4). The kinetic traces of both concentration and total absorbance were readily fitted by Eq. (6) (adequate to the system investigated). The changes in photokinetics were quantified by the initial velocity. By varying the radiation intensity of the light source, a proportional (linear) increase of $RK:r_{o,X}^{Lp,∆\lambda }$ , $Fit:r_{o,X}^{Lp,∆\lambda }$ , $Theo:r_{o,A}^{Lp,∆\lambda }$ , and $Fit:r_{o,A}^{Lp,∆\lambda }$ with $P_{0}^{Lp,∆\lambda }$ were evidenced, irrespective of the photomechanism governing the reaction, the lamp profile, the intrinsic and extrinsic parameter values, and/or the differences between $∆\lambda_{Lp}$ , *OSIA*_*act*_, and *OSIA*_*sp*_.

These findings lead to a straightforward kinactinometric methodology for the standardization of an actinometer, $ACT_{1}$ , exposed to the polychromatic light of a lamp, $Lp_{1}$ . Here, we consider the most common situations, *sit*_*1*_ and *sit*_*2*_. These are differentiated by the wavelength span of the lamp toward the actinometer absorption, where ∆λ is used when $∆\lambda_{Lp_{1}}=OSIA_{{act}_{1}}$ , and $∆\lambda^{+}$ is used when $∆\lambda_{Lp_{1}}>OSIA_{{act}_{1}}$ .

(i) Chose a lamp $Lp_{1}$ , the actinometer $ACT_{1}$ , the initial concentration of $ACT_{1}$ , and the irradiated area and volume of the reactor.
(ii) Irradiate the sample with $Lp_{1}$ , at *n* different intensities, $P_{0}^{Lp_{1},∆\lambda ,i}$ (or $P_{0}^{Lp_{1},∆\lambda^{+},i}$ ), with 1≤i≤n

(iii) Determine the values of the $P_{0}^{Lp_{1},∆\lambda ,i}$ (or $P_{0}^{Lp_{1},∆\lambda^{+},i}$ ) by physical actinometry
(iv) Record the *n* traces of the species concentrations or the total absorbance at a selected observation wavelength, $\lambda_{obs}$

(v) Fit each of the traces available by Eq. (6) or Eq. (9)
(vi) Calculate the *n* initial rates ( $r_{o,X}$ or $r_{o,A}$ ), from the respective *n* fitting parameters using Eq. (8) or Eq. (10)
(vii) Draw the linear graph, for example, $r_{o,A}^{Lp_{1},∆\lambda^{+},i}=\alpha_{Lp_{1}}^{∆\lambda^{+}}\;P_{0}^{Lp_{1},∆\lambda^{+},i}$ , and record the equation of the line (the slope of the line, e.g., $\alpha_{Lp_{1}}^{∆\lambda^{+}}$ , is dimensionless and has the sign of the initial rate).
(viii) Work out the unknown intensity $P_{0}^{Lp_{1},∆\lambda^{+},unk}$ (or $P_{0}^{Lp_{1},∆\lambda ,unk}$ ) of $Lp_{1}$ from the equation of the line obtained in point vii) together with the corresponding $r_{o}^{Lp_{1},∆\lambda^{+},i}$ (or $r_{o}^{Lp_{1},∆\lambda ,i}$ ) determined by the fitting of the $ACT_{1}$ trace that corresponds to $P_{0}^{Lp_{1},∆\lambda^{+},unk}$ (or $P_{0}^{Lp_{1},∆\lambda ,unk}$ ), as

$$P_{0}^{Lp_{1},∆\lambda^{+},unk}=\frac{r_{o,A}^{Lp_{1},∆\lambda^{+},unk}}{\alpha_{Lp_{1}}^{∆\lambda^{+}}}\left(or\;P_{0}^{Lp_{1},∆\lambda ,unk}=\frac{r_{o,A}^{Lp_{1},∆\lambda ,i}}{\alpha_{Lp_{1}}^{∆\lambda }}\right).$$ (13)

(ix) if the *OSIA* and the profile of the lamp are known, calculate the *n* $P_{0}^{Lp_{1},∆\lambda^{+},i}$ from $P_{0}^{Lp_{1},∆\lambda ,i}$ using Eq. (15) (*vide infra*) and Eq. (13) (and *vice versa*, $P_{0}^{Lp_{1},∆\lambda ,i}$ from $P_{0}^{Lp_{1},∆\lambda^{+},i}$ , Eq. (13) and Eq. (15).

The linearity of the plots in both cases where ∆λ and $∆\lambda^{+}$ are considered, can be explained by the following. The total number of photons entering the reactor can be viewed as a sum, $P_{0}^{Lp_{1},∆\lambda^{+},i}=P_{0}^{Lp_{1},∆\lambda ,i}+P_{0}^{Lp_{1},\left(∆\lambda^{+}-∆\lambda \right),i}$ , with $P_{0}^{Lp_{1},\left(∆\lambda^{+}-∆\lambda \right),i}$ being the extra number of photons not absorbed by the actinometer (assuming $∆\lambda^{+}>∆\lambda$ ). The initial rate values obtained by the fitting of the traces of the two cases are invariant, $r_{o,A}^{Lp_{1},∆\lambda^{+},i}=r_{o,A}^{Lp_{1},∆\lambda ,i}$ , that is, not affected by the presence of the extra photons, $P_{0}^{Lp_{1},\left(∆\lambda^{+}-∆\lambda \right),i}$ , because the reaction is induced by the same number of photons covering the $Lp_{1}/ACT_{1}$ *OSIA*. In addition, it is obvious that an increase recorded on $P_{0}^{Lp_{1},\left(∆\lambda^{+}-∆\lambda \right),i}$ is proportional to the increase of $P_{0}^{Lp_{1},∆\lambda ,i}$ because both belong to the same lamp, such as $P_{0}^{Lp_{1},∆\lambda ,i+1}/P_{0}^{Lp_{1},∆\lambda ,i}=P_{0}^{Lp_{1},\left(∆\lambda^{+}-∆\lambda \right),i+1}/P_{0}^{Lp_{1},\left(∆\lambda^{+}-∆\lambda \right),i}$ . Therefore, the ratio $P_{0}^{Lp_{1},\left(∆\lambda^{+}-∆\lambda \right),i}/P_{0}^{Lp_{1},∆\lambda ,i}=\beta_{Lp_{1}}^{∆\lambda^{+}/∆\lambda }$ is dimensionless and necessarily constant irrespective of the actual intensity of the radiation ( 1≤i≤n ). The $\beta_{Lp_{1}}^{∆\lambda^{+}/∆\lambda }$ factor is a property of the lamp and the actinometer used. With $P_{0}^{Lp_{1},\left(∆\lambda^{+}-∆\lambda \right),i}=\beta_{Lp_{1}}^{∆\lambda^{+}/∆\lambda }\;P_{0}^{Lp_{1},∆\lambda ,i}$ , we derive from the above relationship, $P_{0}^{Lp_{1},∆\lambda^{+},i}=P_{0}^{Lp_{1},∆\lambda ,i}+\beta_{Lp_{1}}^{∆\lambda^{+}/∆\lambda }\;P_{0}^{Lp_{1},∆\lambda ,i}=\left(1+\beta_{Lp_{1}}^{∆\lambda^{+}/∆\lambda }\right)\;P_{0}^{Lp_{1},∆\lambda ,i}$ .

Hence, for a given lamp, $Lp_{1},$ we have

$$r_{o,A}^{Lp_{1},∆\lambda^{+},i}=r_{o,A}^{Lp_{1},∆\lambda ,i}=\alpha_{Lp_{1}}^{∆\lambda^{+}}\;P_{0}^{Lp_{1},∆\lambda^{+},i}=\alpha_{Lp_{1}}^{∆\lambda^{+}}\;\left(1+\beta_{Lp_{1}}^{∆\lambda^{+}/∆\lambda }\right)\;P_{0}^{Lp_{1},∆\lambda ,i}=\alpha_{Lp_{1}}^{∆\lambda }\;P_{0}^{Lp_{1},∆\lambda ,i}$$ (14)

and

$$\alpha_{Lp_{1}}^{∆\lambda^{+}}=\frac{\alpha_{Lp_{1}}^{∆\lambda }}{1+\beta_{Lp_{1}}^{∆\lambda^{+}/∆\lambda }}.$$ (15)

Such an analysis clearly shows that actinometric measurements can be performed whether or not the profile of the lamp partially covers the electronic absorption spectrum of the actinometer. When the lamp emission profile is equal to or less than the actinometer’s absorption wavelength span, $∆\lambda ≅∆\lambda^{+}$ , $P_{0}^{Lp_{1},\left(∆\lambda^{+}-∆\lambda \right),i}≅0$ , the factor $\beta_{Lp_{1}}^{∆\lambda^{+}/∆\lambda }\ll 1$ , and $\alpha_{Lp_{1}}^{∆\lambda }=\alpha_{Lp_{1}}^{∆\lambda^{+}}$ . Otherwise, when $∆\lambda <∆\lambda^{+}$ , we have $\alpha_{Lp_{1}}^{∆\lambda^{+}}<\alpha_{Lp_{1}}^{∆\lambda }$ . This reasoning holds as well if $∆\lambda^{+}$ represents the *OSIA* of the actinometer, and ∆λ represents that of the investigated species as set out for *sit*_*1*_. This proves that $r_{o,A}^{Lp_{1},∆\lambda^{+},i}$ is linearly proportional to both $P_{0}^{Lp_{1},∆\lambda^{+},i}$ and $P_{0}^{Lp_{1},∆\lambda ,i}$ . Knowing one proportionality factor (e.g., $\alpha_{Lp_{1}}^{∆\lambda^{+}}$ ) will allow working out the value of the other ( $\left.\alpha_{Lp_{1}}^{∆\lambda }\right)$ if the value of $\beta_{Lp_{1}}^{∆\lambda^{+}/∆\lambda }$ is known. Knowing $\alpha_{Lp_{1}}^{∆\lambda }$ allows the determination of $P_{0}^{Lp_{1},∆\lambda ,i}$ (and vice versa). $\beta_{Lp_{1}}^{∆\lambda^{+}/∆\lambda }$ can be determined during the actinometer standardization if the lamp’s profile, $P_{0}^{\lambda_{irr}}$ , is known (because the absorption spectrum of $ACT_{1}$ must be known).

The demonstrated usefulness of the factors $\beta_{Lp_{1}}^{∆\lambda^{+}/∆\lambda }$ in kinactinometry recommends that the numeric values of lamp profiles ( $P_{0}^{\lambda }$ against λ ) be published. The construction of a database of those lamp profiles is acutely needed in this context. Such a database would be most helpful to the community for expanding the library of available, accurate, and handy actinometers.

**FIGURE 12:**
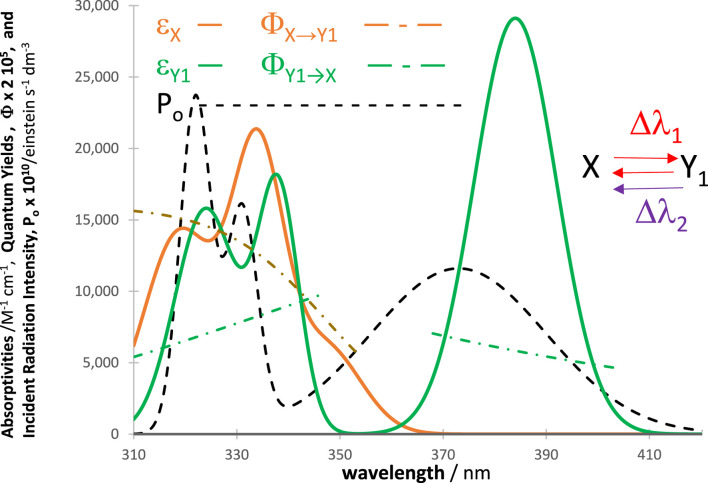
Profiles of absorptivities, quantum yields, and light-source intensities of a typical reaction of diarylethenes. The photomechanism ( $m_{5}$ in Scheme 1) includes a concomitant photoreversible reaction when irradiated in one section of wavelengths ( ${∆\lambda }_{1}$ between 310 nm and 350 nm, where both the reactant and photoproduct absorb), and a back reaction when irradiated in another region (Δλ_2_ = 350 nm–420 nm, where only the photoproduct, $Y_{1}$ , absorbs). The reactive system is continuously under $∆\lambda^{+}=∆\lambda_{1}+∆\lambda_{2}$ irradiation.

The kineactinometric methodology presented above applies to the most commonly used organic actinometers in the field ( $m_{1}to\;m_{4}$ , Scheme 1). For illustration, Figure 12 shows the properties of a photoreversible system involving three reaction steps ( $m_{5}$ in Scheme 1). When subjected to irradiation, these reaction steps are variably sensitive to the different spans covered by the lamp profile. Irradiation light occurring over $∆\lambda_{1}$ (e.g., 310–350 nm, in Figure 12) causes a photoreversible reaction between the reactant and the photoproduct, whereas that spanning $∆\lambda_{2}$ (e.g., 350–420 nm, in Figure 12) induces a back reaction of the photoproduct only. The reactive system is continuously subjected to $∆\lambda^{+}=310-420\;nm$ , where the reactant is only sensitive to $∆\lambda =∆\lambda_{1}$ and the photoproduct to both $∆\lambda_{1}$ and $∆\lambda_{2}$ ( $∆\lambda^{+}=∆\lambda_{1}+∆\lambda_{2}$ ). This reactive system mimics that of diarylethenes, which in real-life situations would simultaneously be exposed to UV ( $∆\lambda_{1}$ ) and visible ( $∆\lambda_{2}$ ) irradiation. As far as we are aware, the photokinetics of diarylethenes has never been studied when subjected to concomitant irradiation by UV and visible light. In addition, even though a diarylethene derivative was proposed for visible light actinometry (Roibu et al., 2018; Maafi and Al Qarni, 2022a), there are no known equivalent investigations of such a system when subjected to concomitant UV and visible light.

**FIGURE 13:**
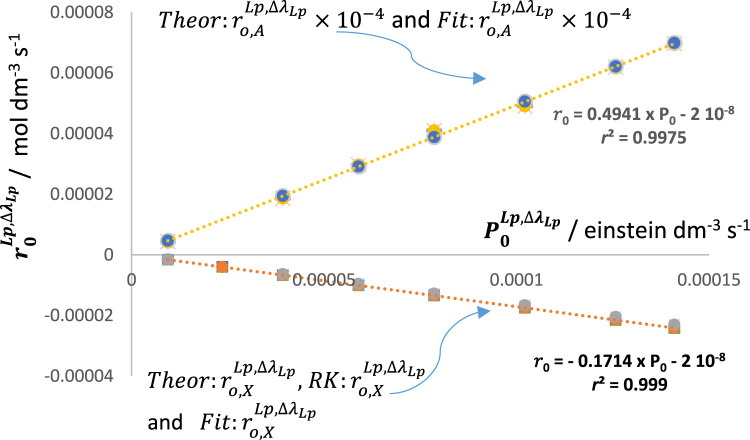
Linear relationships between the initial velocity values worked out from the traces of both the reactant ( $r_{o,X}^{Lp,∆\lambda^{+}}$ ) and total absorbance ( $r_{o,A}^{Lp,∆\lambda^{+}}$ ) with the incident radiation intensity ( $P_{o}^{Lp,∆\lambda^{+}}$ ).

The RK-generated traces for the photosystem depicted in Figure 12 were fitted by an Eq. (6) (and Eq. (9)) that encompasses a single mono- Φ -order term. Figure 13 shows that the reaction is a good actinometer as both its initial reactant-rate ( $Theo:r_{\left(0,X\right)}^{\left(Lp,∆\lambda^{+}.\right)}$ , $RK:r_{\left(0,X\right)}^{\left(Lp,∆\lambda^{+}\right)}$ and $Fit:r_{\left(0,X\right)}^{\left(Lp,∆\lambda^{+}\right)}$ ), and the initial rates of the total absorbance trace ( $Theo:r_{\left(0,A\right)}^{\left(Lp,∆\lambda^{+}\right)}$ , and $Fit:r_{\left(0,A\right)}^{\left(Lp,∆\lambda^{+}\right)}$ ) are equally linearly proportional to $P_{0}^{\left(Lp,∆\lambda^{+}\right)}$ (with $∆\lambda^{+}=310-420\;nm$ ).

Practically, in general, actinometry will only deliver the values $P_{0}^{Lp,∆\lambda^{+},i}$ at *i* radiation intensities but not the corresponding numbers of photons serving the reactant photoconversion, $P_{0}^{Lp,∆\lambda ,i}$ (because, technically, it is not usually possible to selectively filter the light of the lamp to the section of wavelengths desired for the experiment, for example, $∆\lambda =∆\lambda_{1}$ in Figure 12). In this case, $P_{0}^{Lp,∆\lambda ,i}$ can be precisely determined using the framework developed above because the lamp profile is known in our example (Figure 12). The factor $\beta_{Lp}^{∆\lambda^{+}/∆\lambda }=P_{0}^{Lp,\left(∆\lambda^{+}-∆\lambda \right),i}/P_{0}^{Lp,∆\lambda ,i}$ is calculated as equal to 1.49 for Figure 12. From there, the series of $P_{0}^{Lp,∆\lambda ,i}$ values are obtained from Eq. (14) as $P_{0}^{Lp,∆\lambda ,i}=P_{0}^{Lp,∆\lambda^{+},i}/\left(1+\beta_{Lp}^{∆\lambda^{+}/∆\lambda }\right)$ . In addition, the $\beta_{Lp}^{∆\lambda^{+}/∆\lambda }$ can be used to calculate the value of $\alpha_{Lp}^{∆\lambda }$ from Eq. 15 and the slope ( $\alpha_{Lp}^{∆\lambda^{+}}=-\;0.1714$ ) of the initial rate vs. intensity in Figure 13, as $\alpha_{Lp}^{∆\lambda }=-\;0.427$ . As predicted, for the present case, because $∆\lambda <∆\lambda^{+}$ , and $\beta_{Lp}^{∆\lambda^{+}/∆\lambda }>1$ , the value of $\alpha_{Lp}^{∆\lambda^{+}}$ is bigger than that of $\alpha_{Lp}^{∆\lambda }$ (or $-\;\alpha_{Lp}^{∆\lambda^{+}}<-\;\alpha_{Lp}^{∆\lambda }$ ). This procedure also applies when a plot of $r_{o,A}^{Lp,∆\lambda^{+}}$ vs. $P_{0}^{Lp,∆\lambda^{+}}$ is considered. Accordingly, these results promote diaryethenes (and, in general, photochromic materials) to become excellent actinometers with large dynamic ranges covering both UV and visible regions.

Henceforward, and beyond the series shown in Scheme 1, the present kinactinometric methodology may be applied to any photoreactive system (provided that it involves a single reactant). This represents an unprecedented performance in actinometry.

For practicality, it might be useful to consider the whole set of photons produced by the lamp for actinometric standardization ( $P_{0}^{Lp_{1},∆\lambda_{Lp_{1}},i}$ measured over the full emission domain, $∆\lambda_{Lp_{1}}$ , of the lamp, and not those absorbed by the actinometer over the *OSIA*, ∆λ ). The advantage here is to provide that information about the lamp ( $P_{0}^{Lp,∆\lambda_{Lp}}$ ) to the experimentalist. In this particular case, the number of photons collected is equivalent to the number provided by the ferrioxalate actinometer, a consideration explored in Section 3.11.

### 3.10 Specificity of the lamp, actinometer, and reaction conditions

A standardized actinometer is a powerful tool in photokinetics that, according to the general assumption, can be used to determine the photons amount, $P_{0}$ , of any light source. Unfortunately, kinactinometry states otherwise. Indeed, our discussion in the previous sections indicated that the general Eq. (4) becomes specific when applied to a given reaction. This stems from the uniqueness of the reaction attributes and the experimental conditions under which that reaction is performed. Hence, it becomes evident that the actinometer, lamp, and reaction conditions are unique for the specific *OSIA* of the lamp/reaction. In other words, an actinometer $ACT_{1}$ standardized with lamp $Lp_{1}$ , as set out in Section 3.9, cannot be employed to determine the unknown intensity of another lamp, $Lp_{2}$ , to which $ACT_{1}$ is subjected. This is true because the light profiles of these lamps are different (assuming all other conditions are the same), which leads to $\alpha_{Lp_{1}}^{∆\lambda }$ being different from $\alpha_{Lp_{2}}^{∆\lambda }$ (if the *OSIA* is assumed to be the same for $ACT_{1}$ and the two lamps). In real-life situations, the lamp profiles and the *OSIA*s of the actinometer will most likely differ.

Likewise, if different actinometers are exposed to the same lamp and deliver the same total number of photons in each case, it is expected that the resulting actinometric calibration lines of the actinometers will be different due to the actinometers’ attributes (their intrinsic parameters $\Phi^{\lambda_{irr}}$ and $\varepsilon^{\lambda_{irr}}$ ) being different.

Another observation has an additional indirect but still important consequence on experimental photochemistry. Indeed, it is common to consider that the value obtained by chemical actinometry for the radiation intensity entering the reactive medium is to be directly used for the calculations of photokinetic parameters of the investigated system. This implicitly supposes two assumptions. On the one hand, the number of photons determined by using a given actinometer is valid for the lamp and reaction used, and on the other hand, the same number of photons will be absorbed by both the actinometer and the reaction being studied, that is, $OSIA_{sp}=OSIA_{act}$ , (Zalazar et al., 2005). These assumptions are not necessarily consistent in all cases, as discussed in the previous section.

Hence, we must admit that the classical method of making actinometric measurements using a given actinometer that was standardized by a specific lamp is not necessarily useful for investigating the reaction at hand. Consequently, one concludes that the best actinometer would be the investigated reaction itself. However, this remains a dream target for actinometry under polychromatic light.

To turn the reactant into an actinometer (let us label it X−ACT ), we can use the methodology developed above in Section 3.9 (where X−ACT stands for $ACT_{1}$ in that procedure requiring physical actinometry). Otherwise, if a chemical actinometer $ACT_{1}$ is required to determine the radiation intensity, then it is mandatory that the *OSIA* of the standardized chemical actinometer ( $ACT_{1}$ ), for the lamp used, is larger than that of X−ACT , and the $\beta_{Lp}^{∆\lambda^{+}/∆\lambda }$ of X−ACT is known. By applying the method described in Section 3.9, it will be possible to construct the plots $P_{0}^{Lp_{1},∆\lambda_{Lp},i}$ vs. $r_{0,X}^{Lp,∆\lambda^{+}}$ and then $P_{0}^{Lp,∆\lambda ,i}$ vs. $r_{0,X}^{Lp,∆\lambda^{+}}$ for X−ACT , meaning that X−ACT becomes an actinometer. Such a procedure can only be developed if the profile of the lamp is known, again indicating the usefulness of constructing a database for lamp profiles ( $P_{0}^{\lambda_{irr}}=f\left(\lambda_{irr}\right)$ ).

### 3.11 Some observations relative to the ferrioxalate actinometer

In principle, the non-universality of actinometers discussed in the previous section should also apply to ferrioxalate actinometry. In this case, the number of photons recorded by this actinometer is given by the number of ferrous ions produced in solution (via photoreduction of the ferric ions initially present in the medium). However, because the number of ferrous ions produced necessarily depends on both the wavelength-dependent quantum yield of this actinometer (Montalti et al., 2020) and on the profile of the actual lamp used, it is predictable that two different lamps will most likely generate different numbers of ferrous ions. Incidentally, such two hypothetical lamps are most likely different from the light sources used in the original study (Hatchard and Parker, 1956) to calibrate this actinometer. In any case, in ferrioxalate actinometry, the number of photons absorbed by this actinometer is experimentally determined by a global number of ferrous ions produced in solution.

Usually, ferrioxalate actinometry is used to determine the number of photons reaching the actinometric solution, in order to determine the quantum yield of the molecule (reactant) investigated by the use of the differential quantum yield expression.

The number of ferrous ions produced in a ferrioxalate solution will correspond to the photons emitted by the lamp over its whole spectral range if unfiltered (except, perhaps, for λ>500 nm , where it does not absorb). This would be true for almost all classical unfiltered lamps that were traditionally used in experimental photochemistry (filtered lamps and LED lights might be exceptions when $∆\lambda^{+}=∆\lambda$ ).

Each time an unfiltered lamp emission domain is larger than the absorption spectrum of the species studied, the measured number of photons is higher than that corresponding to the photons absorbed by that species. So, for unfiltered lamps, ferrioxalate actinometry measures $P_{0}^{Lp,∆\lambda^{+}}$ and not $P_{0}^{Lp,∆\lambda }$ . This observation invalidates the evaluation of a reactant quantum yield (when polychromatic light is used) by the differential quantum yield equation using $P_{0}^{Lp,∆\lambda_{Lp}}$ , as practiced in the literature. This is because such evaluated quantum yield values must result in an underestimation of the true quantum yield value because $P_{0}^{Lp,∆\lambda^{+}}$ determined by ferrioxalate actinometry is most likely higher than $P_{0}^{Lp,∆\lambda }$ .

The latter observation also discards an implicit assumption belonging to the ferrioxalate actinometry procedure, by which it is held that all photons absorbed by this actinometer are also absorbed by the species studied (whose quantum yield is sought). The inequality $P_{0}^{Lp,∆\lambda^{+}}=P_{0}^{Lp,∆\lambda_{Lp}}>P_{0}^{Lp,∆\lambda }$ makes the above assumption an invalid assertion (Lente, 2015). This also holds for the cases when a high concentration of the reactant is used.

In addition, one can relay another observation about the ferrioxalate procedure: the necessary consideration of the investigated species’ quantum yield invariance with wavelength in order to apply the differential quantum yield equation (as discussed in Section 3.12). This remains an approximation until otherwise proven experimentally (which can only be implemented by using strictly monochromatic lights at different wavelengths where the species absorbs (Maafi, 2023)). It is very rare that published works perform screening of irradiations at individual wavelengths preceding investigations with polychromatic light. Therefore, an imposed Φ -invariance assumption might lead to inaccuracy of the determined quantum yield values.

As a matter of fact, the above observations are most often overlooked in the literature. Taking them into account and specifying the details of the experiment becomes a necessity for future research.

The kinactinometry methodology presented in Sections 3.9, 10, and 11 avoids such limitations of ferrioxalate and classical actinometries and allows determination of both the number of photons absorbed by the organic actinometer within its *OSIA*, as well as the total number of photons emitted by the lamp that reached the actinometric solution.

### 3.12 Polychromatic light irradiation and quantum yield

It is interesting to explore the possibilities of evaluating a quantum yield for a reaction subjected to polychromatic light. In principle, such an evaluation should not be envisaged according to the IUPAC (Braslavsky, 2007; Braslavsky et al., 2011). The IUPAC definition of *quantum yield* considers that the term is valid only for strictly monochromatic irradiation. Instead, for polychromatic light within a given wavelength range, the IUPAC indicates that either terms of *photonic yield* or *quantum efficiency* are more convenient. In this context, the quantum yield refers to the number of monochromatic photons absorbed, whereas the photonic yield refers to the number of monochromatic photons over a given ∆λ -domain arriving at the internal surface of the irradiated window of the reactor. This definition of the photonic yield (referring to the number of photons incident on a surface but not absorbed) is in violation of the “first law of photochemistry.” It was suggested that in this context, the photonic yield may be interpreted as the amount of energy used for a reaction referred to the energy impinging on the surface (Braslavsky et al., 2011). However, it is important to underline that the photonic yield formula (*vide infra* Eq. (18)) has neither been derived analytically from the rate law nor has it been deduced from empirical data. Hence, the photonic yield may globally be viewed as a way to estimate the reactivity of a photosystem relative to the number of photons reaching the reactive medium.

From the point of view of photokinetics, a single value for the quantum yield when the light is polychromatic does not make physical sense when the quantum yield is wavelength dependent. Furthermore, Eq. (4) shows the complexity of that issue, where an individual rate equation of a given species $Y_{j}$ may involve the wavelength-dependent quantum yields of several species. Eq. (16) (worked out from Eq. (4)) gives the general expression of the quantum yield of the initial reaction step, $X\to Y_{1}$ , if the latter is assumed to be wavelength-invariant, $\Phi_{X\;\to \;Y_{1}}^{\lambda_{irr}}=\Phi_{X\;\to \;Y_{1}}$ .

$$\Phi_{X\;\to \;Y_{1}}=-\frac{\frac{{dC}_{X}^{∆\lambda }\left(t\right)}{dt}}{\sum_{\lambda_{irr}=\lambda_{a}}^{\lambda_{b}}\left(P_{aX}^{\lambda_{irr}}\right)}-\frac{\sum_{\lambda_{irr}=\lambda_{b}}^{\lambda_{a}}\left(\;\sum_{2}^{\alpha }\Phi_{X\to Y_{j}}^{\lambda_{irr}}\;P_{aX}^{\lambda_{irr}}\right)}{\sum_{\lambda_{irr}=\lambda_{a}}^{\lambda_{b}}\left(P_{aX}^{\lambda_{irr}}\right)}+\frac{\sum_{\lambda_{irr}=\lambda_{a}}^{\lambda_{b}}\left(\sum_{1}^{\beta }\Phi_{Y_{j}\to X}^{\lambda_{irr}}\;P_{aY_{j}}^{\lambda_{irr}}\;\right).}{\sum_{\lambda_{irr}=\lambda_{a}}^{\lambda_{b}}\left(P_{aX}^{\lambda_{irr}}\right)}$$ (16)

Eq. (16) resembles a differential quantum yield equation but considerably differs from those classically proposed for that purpose for reactions under monochromatic (Braslavsky, 2007) or polychromatic (Braslavsky et al., 2011) light. It is, however, close to that proposed previously for the general quantum yield equation of the same reaction ( $X\to Y_{1}$ ) under monochromatic light (Maafi, 2023). Amending the IUPAC report (Braslavsky et al., 2011) with Eq. (16) and the assumptions leading to its derivation might be useful.

Despite the simplifying conditions considered here ( $\Phi_{X\;\to \;Y_{1}}^{\lambda_{irr}}=\Phi_{X\;\to \;Y_{1}}$ ), this formulation (Eq. (16)) cannot possibly be evaluated experimentally. Alternatively, it may be calculated if all the parameter values are known for all wavelengths of the ∆λ -domain (this includes the quantum yields $\Phi_{X\to Y_{j}}^{\lambda_{irr}}$ and $\Phi_{Y_{j}\to X}^{\lambda_{irr}}$ , which, incidentally, would not justify that only $\Phi_{X\;\to \;Y_{1}}$ is not known). Unfortunately, the knowledge of all these parameters is not usually affordable in real-life situations.

Photokinetically, under the conditions $\Phi_{X\;\to \;Y_{1}}^{\lambda_{irr}}=\Phi_{X\;\to \;Y_{1}}$ when the reactant only depletes to form $Y_{1}$ , $X\;\to \;Y_{1}$ , we can propose Eq. (17) to accurately determine the quantum yield whenever the trace of the reactant and $P_{0}^{\lambda_{irr}}$ are available. This also requires fitting the trace with an adequate Eq. (6) and determining $Fit:r_{0,X}^{Lp,∆\lambda }.$

$$\Phi_{X\;\to \;Y_{1}}=-\frac{Fit:r_{0,X}^{Lp,∆\lambda }}{\;\sum_{\lambda_{irr}=\lambda_{a}}^{\lambda_{b}}\left(P_{0}^{\lambda_{irr}}\;\left(1-10^{-\;A_{X}^{\lambda_{irr}}\left(0\right)}\right)\;\right)}.$$ (17)

For the general case ( $\Phi_{X\;\to \;Y_{1}}^{\lambda_{irr}}\neq \Phi_{X\;\to \;Y_{1}}$ ), however, there is no analytical, single expression able to evaluate the efficiency of a photoreaction under polychromatic light.

The commonly used photonic yield ( PHYD ) is measured as the ratio of the reacted reactant concentration at time t (when the photoconversion of X is targeted) to the total number of photons entering the reactor in the same laps of time. If the irradiation corresponds to the *OSIA*_*X*_, we have

$${PHYD}_{X}^{Lp,∆\lambda }\left(t\right)=\frac{C_{X}^{Lp,∆\lambda }\left(0\right)-C_{X}^{Lp,∆\lambda }\left(t\right)}{P_{0}^{Lp,∆\lambda }\;t},$$ (18)

Or, when PHYD is measured for a particular end product, $Y_{end}$ ,

$${PHYD}_{Y_{end}}^{Lp,∆\lambda }\left(t\right)=\frac{C_{Y_{end}}^{Lp,∆\lambda }\left(t\right)}{P_{0}^{Lp,∆\lambda }\;t}.$$ (19)

PHYD is dimensionless according to the following dimension analysis, $\left[PHYD\right]=\left(mol\times L^{-1}\right)\;/\left(einstein\times dm^{-3}\times s^{-1}\times s\right)=1$ .

The $P_{0}^{Lp,∆\lambda }$ can be determined by chemical actinometry using a standardized actinometer if $\beta_{Lp}^{∆\lambda^{+}/∆\lambda }$ is known (as discussed in Section 3.8). The concentration of either reactant or selected final product is obtained by routine analytical techniques, and t is the irradiation time.

Because the incident radiation intensity entering the reactor can be determined either as $P_{0}^{Lp,∆\lambda }$ or $P_{0}^{Lp,∆\lambda^{+}}$ , the value of ${PHYD}^{Lp,∆\lambda^{+}}$ will be lower than that obtained for ${PHYD}^{Lp,∆\lambda }$ for the same reaction. It is then mandatory to specify, in any investigation, which of $P_{0}^{Lp,∆\lambda }$ or $P_{0}^{Lp,∆\lambda^{+}}$ has been used to calculate the PHYD by Eqs (18) and (19).

Remarkably, the photonic yield of a reactant (or a product, Eq. (18) and Eq. (19)) is time-dependent because the reaction of X (or $Y_{end}$ ) is dependent on time and on the reactivity of the other absorbing species in the medium. The timely change of the reactant or the end-product concentrations ( $C_{X}^{Lp,∆\lambda }\left(t\right)$ or $C_{Y_{end}}^{Lp,∆\lambda }\left(t\right)$ ) are necessarily non-linear, whereas the number of photons delivered to the reactive system ( $P_{0}^{Lp,∆\lambda }\;t$ ) is linear with time, hence the non-linearity of ${PHYD}_{X}^{Lp,∆\lambda }$ (or ${PHYD}_{Y_{end}}^{Lp,∆\lambda }$ ) with time. It is, therefore, required to indicate the sampling time at which PHYD is calculated. ${PHYD}_{X}^{Lp,∆\lambda }$ and ${PHYD}_{Y_{end}}^{Lp,∆\lambda }$ are, respectively, monotonical decreasing and increasing functions of reaction time until times ( $t_{end}$ ) where the reactions end, that is, $C_{X}^{Lp,∆\lambda }\left(t_{end,X}\right)$ and $C_{Y_{end}}^{Lp,∆\lambda }\left(t_{end,Y_{end}}\right)$ have constant values, then, with increasing time, ${PHYD}_{Y_{end}}^{Lp,∆\lambda }$ should decrease until reaching a zero value. Incidentally, because the non-linearity of PHYD has not been emphasized in the IUPAC document (Braslavsky et al., 2011), it will be helpful to amend that reference document accordingly.

Similarly, PHYD (Eq. (18) and Eq. (19)) is expected to vary with radiation intensity (e.g., $P_{0}^{Lp,∆\lambda }$ ) (Reiß et al., 2021), with initial reactant concentration (Jakusová et al., 2014; Menzel et al., 2021), and with any other change in the medium composition (e.g., presence of *SPM*s) and/or experimental conditions (e.g., change of $l_{irr}$ ). This makes PHYD a relative quantity that should be used with caution when comparing reactive systems or the effect of reaction conditions.

In line with our use of initial velocity as a metric for quantification of photokinetic events, let us introduce Eq. (20) for the evaluation of the efficiency of a photoreaction under polychromatic light ( PY ).

$${PY}_{X}^{Lp,∆\lambda^{+}}=-\frac{r_{0,X}^{Lp,∆\lambda^{+}}}{P_{0}^{Lp,∆\lambda^{+}}}\left(or\;{PY}_{X}^{Lp,∆\lambda }=-\frac{r_{0,X}^{Lp,∆\lambda }}{P_{0}^{Lp,∆\lambda }}\right).$$ (20)

PY is a dimensionless, positive constant that reflects the impact due to any changes in the experimental conditions of the reaction (e.g., $P_{0}^{Lp,∆\lambda_{Lp}}$ ; $C_{X}^{Lp,∆\lambda_{Lp}}\left(0\right)$ ; SPM *,* $l_{irr}$ , …etc.). It fulfills the same objectives of PHYD , but in contrast to the latter, it is time-independent, hence increasing the reliability of comparison between investigations. A similar equation can also be devised for the total absorbance trace, where $r_{0,X}$ is replaced by $r_{0,A}$ in Eq. (20) (where the negative sign is discarded if $r_{0,A}$ is positive). This relieves the evaluation of the reaction efficiency from the necessity of acquiring the trace of the reactant. $r_{0,X}$ and $r_{0,A}$ are easily obtained from the fitting of the corresponding traces with adequate Eqs. (6)–(9), respectively. However, PY cannot be expressed for the end products.

In the case of measuring the effect of the variation of the radiation intensity on the reaction efficiency, ${PY}_{X}^{Lp,∆\lambda^{+}}$ is equal to $-\alpha_{Lp}^{∆\lambda^{+}}$ (and ${PY}_{X}^{Lp,∆\lambda }=-\alpha_{Lp}^{∆\lambda }$ ) as given by Eq. (13) (and Figure 13). The pattern of PY variation with initial concentration follows that given in Figure 8. For this particular case, the initial rates are divided by the constant $P_{0}^{Lp,∆\lambda^{+}}$ measured for that experiment. Similarly, the evolution of PY depicts the trend shown in Figure 6 when SPM absorbance is increased.

### 3.13 Does photokinetics work for high initial reactant concentrations?

A concentration is considered high when it falls beyond the higher limit of the linearity range of the species’ calibration graphs (otherwise, the case conforms to both the framework developed in the present study for polychromatic light and that dedicated to monochromatic light (Maafi, 2023)). The obvious consequence of employing a high initial concentration is a deviation from the linearity of the species calibration graph. Such a deviation is proof of the limit of applicability of the Beer–Lambert law and the likelihood of the occurrence of phenomena other than simply a quantitative absorption of the light by the species under study. A full explanation for the observed curvature of the calibration graph is not yet available in the literature. However, several tentative interpretations have been proposed.

Buijs and Maurice (1969) proposed that such behavior had physical, chemical, and/or instrumental causes. The study introduced a procedure to distinguish between these three types of effects but did not derive an alternative to the Beer–Lambert law. The authors have suggested that such deviations from linearity can be attributed to three main causes: the light source being far from monochromatic, the concentrations of analytes being very high, and/or the medium being highly scattering.

One practical attempt focused on mapping the curved shape of the non-linear calibration graph by a Gaussian regression analysis that led to absorbance/absorbent concentration relationships (Young, 2015) but did not investigate possible causes of the curvature. Similarly, more sophisticated mathematical methods, such as random forests, support vector regression, neural networks, PLS, and PCR, have also been applied for such practical purposes (Wu et al., 1996; Santana et al., 2019; Mekonnen et al., 2020; Mamouei et al., 2021).

Recently, a finer analysis using Maxwell’s wave equation derived Beer’s law from dispersion theory and showed it to be a limiting law (Mayerhofer et al., 2019). The indices of absorption and molar attenuation coefficients, including absorption cross sections, were found not to be properties of the material. The latter can, *per se*, not be additive (while they are supposed to be both properties of molecules and additive in the Beer–Lambert law). In this model, the deviation from linearity was found to scale up with oscillator strengths, that is, with local electric fields and nearfield effects. A particular finding of the study indicated that the parameters emerging from this derivation do not exactly match with those of the formulation of the Beer–Lambert law.

A more extensive overview of the deviation of the Beer–Lambert law (Mayerhofer et al., 2020) has advanced an understanding of the optical spectra and their quantitative interpretation based on oscillator positions, strengths, and damping constants. By using the electromagnetic theory, the study showed that it was possible to reach a better quantification of band shift and intensity variation of the absorption spectrum and to conclude that the actual formula of the Beer–Lambert law (proportionality of absorbance with absorbent concentration) is an ideal absorption law that is only valid for relatively low concentration domains (i.e., the linear section of the calibration curve).

In addition to these variations of the absorption coefficients, the possible absorption of the light by other emerging species in the medium due to high concentration (e.g., complexes (Tonnesen, 2004), quenching (Menzel et al., 2021)… etc.), has also been proposed.

In summary, if the concentration value falls outside the linearity range of the calibration graph, then the Beer–Lambert law ceases to apply, and the absorption coefficient values of a species will change from those recorded when its concentration belongs to the linearity range. Unfortunately, often such new values of ε for highly concentrated media are not accessible experimentally. Furthermore, the possible occurrence of other absorbers in the reactive medium (other than the reaction species) adds an extra complication in using the Beer–Lambert law in its classical form. Here, we ought to remember that the Beer–Lambert law only applies to monochromatic light (it is not appropriate when polychromatic light is absorbed by the sample). In addition, even if the literature did not raise the point relative to a change in the quantum yield trend and values with wavelength, it is plausible to consider that such a variation might occur when the concentration is high. By analogy with the alteration of ε when the concentration is high, one might consider postulating that the quantum yield value at a given wavelength might also differ between high- and low-concentration conditions.

A popular procedure in experimental photochemistry bases the evaluation of the reactant quantum yield on the usage of a high reactant concentration (when the reactant depletes by a single reaction step, $X\to Y_{1}$ ). Such a condition is meant to lead the rate of the reactant to be a constant (because $10^{-A_{X}\left(t\right)}≅0$ when $A_{X}\left(t\right)\gg 1$ , as given by Eq. (21)–the latter being a formula usually presented in the literature). This presumably sets the reaction to obey a zeroth-order kinetics (Stranius and Borjesson, 2017; Stadler et al., 2018; Amouroux et al., 2019).

$$\frac{{dC}_{X}\left(t\right)}{dt}=-\;\Phi_{X\to Y_{1}}\;P_{0}\;\left(1-10^{-A_{X}\left(t\right)}\right)=-\;\Phi_{X\to Y_{1}}\;P_{0}.$$ (21)

In this procedure, the quantum yield is then determined by the ratio $\Phi_{X\to Y_{j}}=\left(C_{X}\left(0\right)-C_{X}\left(t_{1}\right)\right)/P_{0}t_{1}$ , where the use of the incident light intensity ( $P_{0}$ ) here is usually justified in the procedure by the fact that, at high concentration, all photons reaching the sample are absorbed by the reactant.

Despite the fact that the above procedure is widely used in the literature, it seems important to clarify that Eq. (21) cannot be established without imposing at least four conditions: i) the rate law applies at high concentration (as it does at low concentrations), ii) the total light absorbed serves specifically the photochemical transformation of the reactant (at least up to time, $t_{1}$ , at which the measurement of the concentration is performed), iii) the quantum yield is considered to be wavelength-independent, and iv) the light is considered monochromatic. A careful consideration of these conditions shows that none is fully justified. Our previous discussion of the non-applicability of the Beer–Lambert law when the concentration is high must impose a modification of the rate law, which invalidates condition i). Because of the possible occurrence of emerging new species in the medium (including photoproducts from the reactant), the fraction of the light absorbed by the reactant is not known with precision and hence, condition ii) is not verified. Of course, the quantum yield might be invariant with wavelength, but this has to be proven experimentally; otherwise, condition iii) cannot be claimed. Finally, condition iv) is easily discarded whenever a monochromator is absent from the experimental setup used for irradiation of the sample. The polychromatic light also requires an integro-differential equation for the rate law (such as Eq. (1)), which does not support the format adopted in Eq. (21) and also discards condition i).

Practically, there is no need to work at high concentrations because Eq. (17) provides such a quantum yield value (under the stated conditions). Otherwise, this value can be determined by successively exposing a low-concentration solution to a monochromatic light (Maafi, 2023). The procedure used in the literature to determine the quantum yield when the initial concentration of the reactant is high corresponds rather to that of a photonic yield ( PHYD , Eq. (18)) than to that of an absolute quantum yield (Eq. (16)). However, whenever an investigation requires the use of high concentration, employing PY (Eq. (20)) is recommended as a means of evaluating the efficiency of the reactant subjected to the specific polychromatic light and other reaction conditions at hand.

Incidentally, the applicability of the high-concentration method for the quantum yield determination, has one serious limitation. Indeed, it is not applicable to those reactive systems that reach a phototostationary state, including the simplest photoreversible reaction (for which other approaches are proposed).

## 4 Conclusion

The Runge–Kutta numerical integration method has shown an excellent aptitude to model the photokinetics of complex photosystems subjected to polychromatic light. It has achieved a comprehensive account of such systems considered in various situations and reaction conditions. Numerical integration represents an efficient tool to prospect and predict photokinetics under both mono- and polychromatic lights.

The general model equation (Eq. (6)) consistently mapped out the photokinetic traces of photoreactions. This represented the first example of this kind in the literature on photochemistry. It proved that photokinetics is well-described by Φ -order kinetics under polychromatic irradiation. It is conjectured that the model equation (Eq. (6)) would also map out the photokinetic traces describing photosystems subjected to uncollimated light, irrespective of the reactor geometry, which is a perspective that would extend the methods proposed in the present article for the widest real-life and engineering studies across many fields of research investigating liquid and solid samples.

The initial rate of the reactant was confirmed as a very useful metric for the quantification of photokinetic behaviors. It was pivotal to evaluating auto-photo-stabilization due to an increasing initial reactant concentration, the reaction slowdown caused by the presence of spectator molecules, increasing the speed of photoreactions with higher radiation intensity, defining of the optimal wavelength region of reactivity (*WROR*), development of actinometers for polychromatic light, and determining the photonic yield ( PY ). These are among the most important and ubiquitous criteria usually determined by experimental photochemistry.

The findings of the present study have shown that quantification of reaction performance in different conditions does not necessarily require the knowledge of the photoreaction mechanism in play. Fitting Eq. (6) or Eq. (9) to the reaction data at hand is sufficient to obtain the metric value (i.e., the initial rate), which in turn allows us to quantitatively assess the reaction or effects of reaction conditions. This is a remarkable tool in photokinetic investigation and an advantage for the study of complex reactions whose mechanisms are often not easily unraveled.

When using polychromatic light, the present study has shown both the inconsistency of seeking the determination of the absolute quantum yield (a constant property of a molecule at a specific, single wavelength) and the utility of determining the photonic yield ( PY ), a relative quantity that depends on the reaction conditions, such as the radiation intensity and the initial concentration.

The present work on polychromatic light complements a previous study on monochromatic light, and both contribute to comprehensively describing and standardizing photokinetics in homogenous media, which is an area that has received little attention in the literature.

Work is now in progress to map out other light sensitive reactions (e.g., photothermal reactions) using a similar strategy.

## Figures and Tables

**Table:** 

$A_{tot}^{\lambda_{irr}}\left(t\right)$	Total absorbance of the reactive medium at wavelength $\lambda_{irr}$ at time t cross $l_{irr}$ (also trace of medium’s total absorbance). $A_{tot}^{\lambda_{irr}}\left(t\right)=A_{tot}^{\lambda_{irr}/\lambda_{irr}}\left(t\right)$
$A_{Y_{j\;or\;j^{\prime }}}^{\lambda_{irr}}\left(t\right)$	Absorbance of species $Y_{j}$ (or $Y_{j^{\prime }}$ ) measured at wavelength $\lambda_{irr}$ and $l_{irr}$ at time t (also trace of $Y_{j}$ (or $Y_{j^{\prime }}$ ) absorbance)
$A_{tot}^{∆\lambda /\lambda_{obs}}\left(t\right)$	Total absorbance of the reactive medium at time t irradiated by lamp Lp and observed at $\lambda_{obs}$ , where the optical path length is $l_{obs}$ (also trace of medium’s total absorbance)
$A_{tot}^{∆\lambda /\lambda_{obs}}\left(\infty \right)$	Total absorbance of the reactive medium at the end of the reaction ( t=∞ )
$A_{{SPM}_{r}}^{\lambda_{irr}}$	Total absorbance of the spectator molecules present in a reactive medium ( 0≤r≤w )
$\alpha_{Lp_{1}}^{∆\lambda }$	Slope of the actinometric plot
$\beta_{Lp_{1}}^{∆\lambda^{+}/∆\lambda }$	A property of the lamp and the actinometer used
$C_{Y_{j}}^{Lp,∆\lambda }\left(t\right)$	The value of $Y_{j}$ concentration at time t (in M or ${mol\;dm}^{-3}$ ), or the kinetic traces of $Y_{j}$
$C_{\infty ,j}^{Lp,∆\lambda }$	Concentration of species $Y_{j}$ at the end of the considered reaction under irradiation with Lp
${cc}_{j}^{∆\lambda }$ **,** ${cc}_{A}^{∆\lambda }$	Pre-exponential factors in, respectively, Eqs.6 and 9, for species $Y_{j}$
∆λ	Wavelength span of the light impinging of the reactor, $∆\lambda =\lambda_{b}-\lambda_{a}$ . In the equations, it represents the *OSIA*
$∆\lambda_{Lp}$	Full wavelength span of the light emitted by the lamp
$∆\lambda_{Act}$	Wavelength span of the lamp’s light overlapping the absorption spectrum of the actinometer
$∆\lambda_{sp}$	Wavelength span of the lamp’s light overlapping the absorption spectrum of the considered species
$∆\lambda_{WROR}$	Wavelength range of optimal reactivity, *WROR*
$∆\lambda^{+}$	Used when $∆\lambda_{Lp}>OSIA_{act}$
e	Exponential function
$\varepsilon_{Y_{j}}^{\lambda_{irr}}$	Absorption coefficient of species $Y_{j}$ at $\lambda_{irr}$ (in $M^{-1}\;{cm}^{-1}$ )
j ***or*** $j^{\prime }$	Index for the species Y ( $Y_{j}$ ), (or $Y_{j^{\prime }}$ , with $j^{\prime }\neq j$ )
$i_{j}$ **or** $i_{A}$	Number of mono- Φ -order terms under the sums of the global equations, Eqs.6 and 8, respectively
$k_{ij}^{∆\lambda }$ **,** $k_{iA}^{∆\lambda }$	Rate constant (in $s^{-1}$ ) of the *ith* regime of species $Y_{j}$ , in Eqs.6 and 9, respectively
**LMA**	Levenberg–Marquardt algorithm
ln	Natural logarithm, base e
Log	Logarithm base 10
Lp	The lamp used to irradiate the sample
$l_{irr}$	Optical path length of the irradiation light from the lamp inside the reactor (in cm )
$l_{obs}$	Optical path length (in cm) of the monitoring light from the spectrophotometer inside the reactor
$\lambda_{irr}$ ***or*** $\lambda_{irr}^{\prime }$	Non-isosbestic irradiation wavelength (in nm , the wavelength of the light beam driving the reaction and different from the wavelengths of isosbestic points)
$\lambda_{obs}$	Observation wavelength at which the medium is monitored
**MatLab**	Software for trace fitting
**NIMs**	Numerical integration methods
$n_{sp}$	Total number of species in the reaction photomechanism, ( $1\leq j\leq n_{sp}$ , with $n_{sp}=8$ for the Φ -shaped reaction mechanism in Scheme 1 of Maafi (2023))
$n_{\Phi }$	Total number of the photochemical reaction steps in the considered photomechanism
$n_{\Phi j}$	The number of photoreaction steps starting or ending at species $Y_{j}$ ( $1\leq n_{\Phi j}\leq n_{\Phi }$ )
***OSIA***	Wavelength span corresponding to the incident irradiation light (from the lamp) that is absorbed by the considered species
$PKF\left(t\right)$	Photokinetic factor of the reaction at hand at time t
$P_{0}^{Lp,∆\lambda }$	Total number of photons entering the reactor, $P_{0}^{Lp,∆\lambda }=\sum_{\lambda_{a}}^{\lambda_{b}}P_{0}^{\lambda_{irr}}$
$P_{0}^{\lambda_{irr}}$	Incident radiation intensity from the lamp at $\lambda_{irr}$ entering the reactor (it is a flux of photons per s per the sample’s irradiated surface $S_{irr}$ and volume $V_{irr}$ ). It is expressed in $einstein\;s^{-1}\;{dm}^{-3}$
$P_{a}^{\lambda_{irr}}\left(t\right)$	Total absorbed light by the reactive medium at time t and at wavelength $\lambda_{irr}$
$P_{a_{Y_{j\;or\;j^{\prime }}}}^{\lambda_{irr}}\left(t\right)$	Fraction of $P_{a}$ that is specifically absorbed by species $Y_{j}$ (or $Y_{j^{\prime }}$ ) at time t and at wavelength $\lambda_{irr}$
PHYD	Photonic yield measured on the basis of concentrations. It is time dependent.
PY	Photonic yield measured on the basis of initial rates. It is a constant.
$\Phi_{Y_{j}\;\to \;Y_{j^{\prime }}}^{\lambda_{irr}}$	Quantum yield of photoreaction step $Y_{j}\;\to \;Y_{j^{\prime }}$ ( $j\neq j^{\prime }$ )
$\Phi_{Y_{j^{\prime }}\;\to \;Y_{j}}^{\lambda_{irr}}$	Quantum yield of photoreaction step $Y_{j^{\prime }}\;\to \;Y_{j}$ ( $j^{\prime }\neq j$ )
***r*** ^**2**^	Squared correlation coefficients
**RK**	Fourth-order Runge–Kutta numerical integration method (RK-NIM)
**RMSD**	Root-mean-square deviation
$r_{Y_{j}}^{Lp,∆\lambda }\left(t\right)$	Rate of species $Y_{j}$ at time t (in $M\;s^{-1}$ ) or rate law for species $Y_{j}$
$r_{\;Y_{0}}^{Lp,∆\lambda }\left(0\right)$	Initial rate of the reactant (in $M\;s^{-1}$ ), ( $r_{\;Y_{0}}^{Lp,∆\lambda }\left(0\right)=r_{X}^{Lp,∆\lambda }\left(0\right)=r_{0,X}^{Lp,∆\lambda }$ )
$r_{Y_{j}}^{\lambda_{irr}}\left(t\right)$	Rate value at wavelength $\lambda_{irr}$ and time t (in $M\;s^{-1}$ ) for species $Y_{j}$ , or rate law for that species
$r_{{0,Y}_{j}}^{\lambda_{irr}}$	Initial reaction rate of species $Y_{j}$ ( $0\leq j\leq n_{sp},$ with for the reactant, $Y_{0}=X$ )
${Fit:r}_{0,Y_{j}}^{Lp,∆\lambda }$	Initial reaction rate of species $Y_{j}$ calculated by using a trace-fitting equation and parameters
${Fit:r}_{0,A}^{Lp,∆\lambda }$	Initial reaction rate of species $Y_{j}$ calculated by using the total absorbance trace-fitting equation and parameters
${RK:r}_{0,Y_{j}}^{Lp,∆\lambda }$	Initial reaction rate of species $Y_{j}$ calculated by RK
${RK:r}_{0,A}^{Lp,∆\lambda }$	Initial reaction rate calculated by RK for the total absorbance trace
${Theo:r}_{0,Y_{j}}^{Lp,∆\lambda }$	Initial reaction rate of species $Y_{j}$ calculated from the rate-law equation (Eq. 4)
${Theo:r}_{0,A}^{Lp,∆\lambda }$	Initial reaction rate calculated from Eq. 10
sp	Index relative to the species
***SPM***	Spectator molecule(s) which are both thermally and photochemically inert
**SSE**	Sum of squares error
$S_{irr}$	Area of the sample (or reactor) that is under irradiation (in ${cm}^{2}$ )
t	Reaction time (in s )
**VBA**	Visual Basic applications
$V_{irr}$	Volume of the sample (or reactor) that is under irradiation (in ${dm}^{3}$ )
$\omega_{ij}^{∆\lambda }$ **,** $\omega_{i,A}^{∆\lambda }$	Pre-logarithmic factors in, respectively, Eqs.6 and 9, for the *i*th regime of species $Y_{j}$
X	Reactant $X=Y_{0}$
$Y_{j}$	Photoproduct, j≠0 ( $1\leq j\leq n_{sp}$ )
$Y_{j^{\prime }}$	Photoproduct linked to $Y_{j}$ by a photochemical step, with $j\neq j^{\prime }$

## Data Availability

The raw data supporting the conclusions of this article will be made available by the authors, without undue reservation.
