# Supplementary material for: On photokinetics under polychromatic light
Source: Front Chem. 2024 Apr 22;12:1367276. doi: 10.3389/fchem.2024.1367276 (PMC11070525; doi:10.3389/fchem.2024.1367276)
Supplement: Supplementary file 1 [file DataSheet1.PDF]

Supplementary materials

For an illustration of the application of the model general equation to experimental data, we use some of the data already published in Ref. (Maafi and Al Qarni, 2022a).

The system studied is the followi diarylethene derivative:

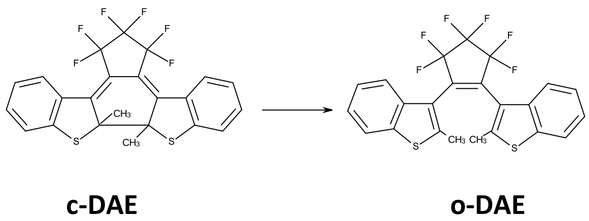

Figure S1: The ring opening photoreaction of diarylethene (*DAE*): 1,2-bis[2-methyl-benzo[*b*]thiophen-3,3,4,4,5,5-hexafluoro-1-cyclopentene.

The derivative is irradiated in solution by lamp that has the following profile:

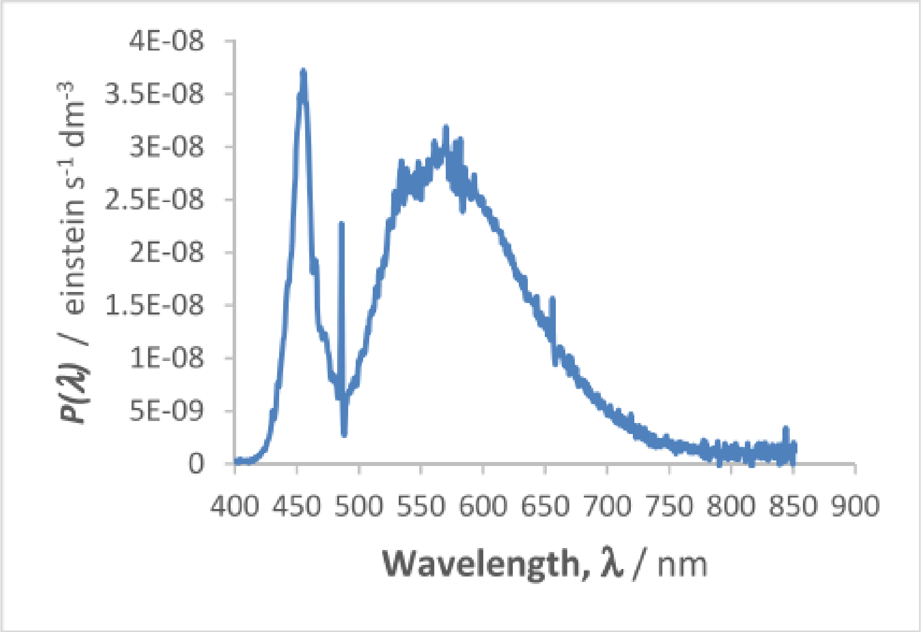

Figure S2. Profile of the light (intensity vs. wavelength) of the LED lamp used in this study. The total intensity, for the shown case, over the range 400–600 nm is 5.95 10^{-6} einstein dm^{-3} s^{-1}.

The absorption spectrum of the derivative recorded a typical decrease as shown in Fig.S3

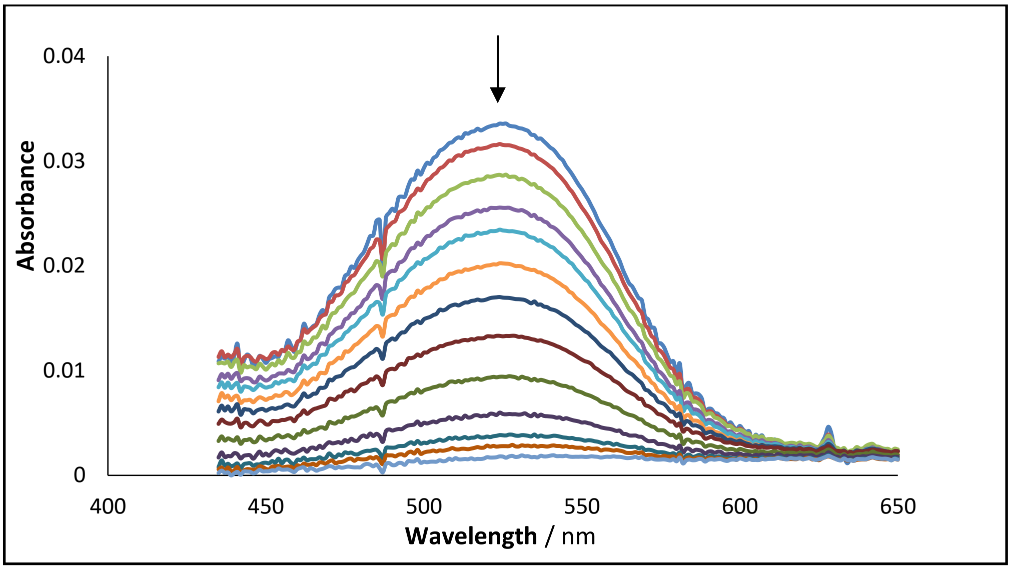

Figure S3. Change of absorption spectra with reaction time

The recorded kinetic data, were fitted by a simplified general model equation (eq.S1):

$$C_{Y_{c-DAE}}^{Lp,\Delta\lambda}(t) = C_{\infty,c-DAE}^{Lp,\Delta\lambda} + \omega_{ic-DAE}^{\Delta\lambda}\ Log\left(1 + cc_{c-DAE}^{\Delta\lambda} e^{-k_{ic-DAE}^{\Delta\lambda} t}\right) \qquad (\boldsymbol{S1})$$

Excellent fittings are shown in Fig.S4 for different initial light intensities of the lamp.

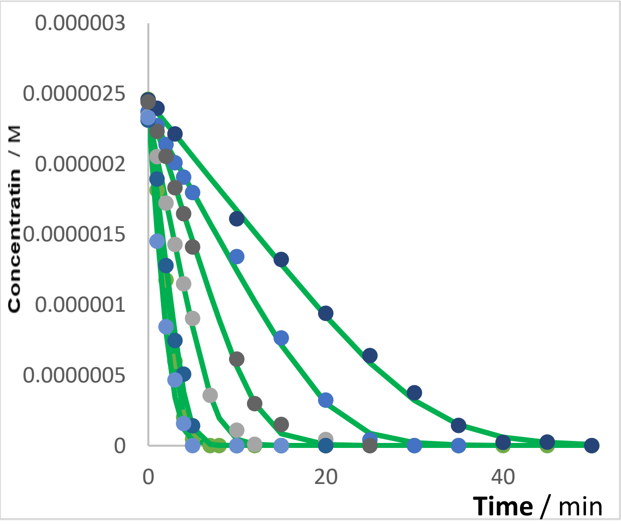

Figure S4: Experimental data (plain circles) of the ring opening photoreaction of diarylethene 1 under polychromatic visible LED-light, and its fitting with the model equation (lines). The solutions were prepared in ethanol at concentrations of *ca*. 4.3 10^{-6} M.

A good linear correlation of initial rate and total light intensity, corroborating the conclusions found in the original paper and confirming the usefulness of the present work (the model equation can serve actinometry).

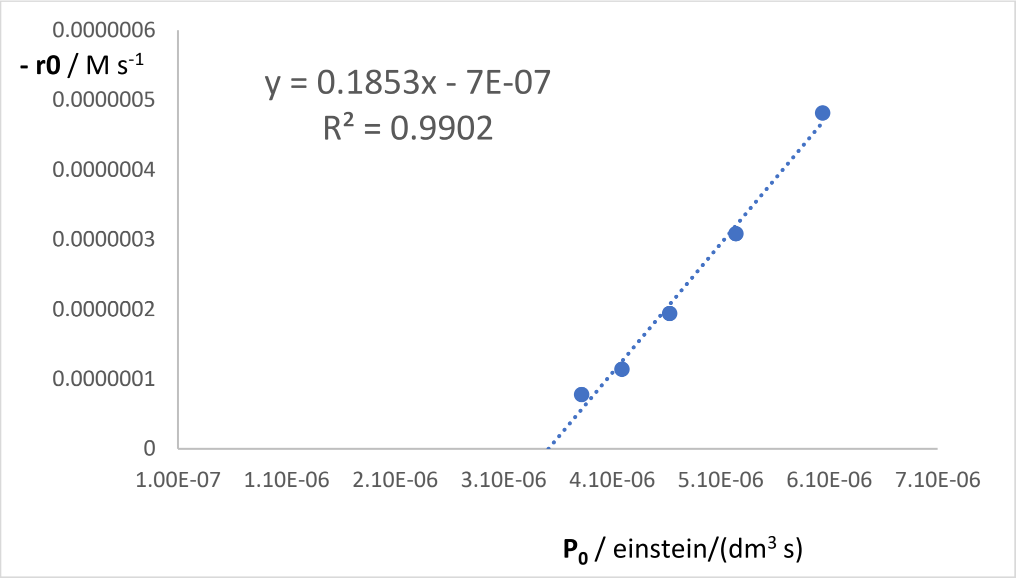

Figure S5. Linear correlation between the total light intensity (P_0) and the initial rate ($- r_0$) of the reactant under irradiation with a polychromatic LED lamp (Fig.S3). The total intensity

Details of the experimental set up and reaction conditions can be found in the original publication (the reader is kindly direct towards that publication for more information on the experiment).
